# Dual stimuli-responsive Fe_3_O_4_ graft poly(acrylic acid)-*block*-poly(2-methacryloyloxyethyl ferrocenecarboxylate) copolymer micromicelles: surface RAFT synthesis, self-assembly and drug release applications

**DOI:** 10.1186/s12951-017-0309-y

**Published:** 2017-10-27

**Authors:** Yuan Wang, Xue-Yin Zhang, Yan-Ling Luo, Feng Xu, Ya-Shao Chen, Yu-Yu Su

**Affiliations:** Key Laboratory of Macromolecular Science of Shaanxi Province, School of Chemistry and Chemical Engineering, Shaanxi Normal University, Xi’an, 710062 People’s Republic of China

**Keywords:** Block copolymers, Magnetic properties, Redox properties, Self-assembly, Stimuli-responsiveness

## Abstract

**Background:**

Stimuli-responsive polymer materials are a new kind of intelligent materials based on the concept of bionics, which exhibits more significant changes in physicochemical properties upon triggered by tiny environment stimuli, hence providing a good carrier platform for antitumor drug delivery.

**Results:**

Dual stimuli-responsive Fe_3_O_4_ graft poly(acrylic acid)-*block*-poly(2-methacryloyloxyethyl ferrocenecarboxylate) block copolymers (Fe_3_O_4_-*g*-PAA-*b*-PMAEFC) were engineered and synthesized through a two-step sequential reversible addition-fragmentation chain transfer polymerization route. The characterization was performed by FTIR, ^1^H NMR, *SEC*, XRD and TGA techniques. The self-assembly behavior in aqueous solution upon triggered by pH, magnetic and redox stimuli was investigated via *zeta* potentials, vibration sample magnetometer, cyclic voltammetry, fluorescent spectrometry, dynamic light scattering, XPS, TEM and SEM measurements. The experimental results indicated that the Fe_3_O_4_-*g*-PAA-*b*-PMAEFC copolymer materials could spontaneously assemble into hybrid magnetic copolymer micromicelles with core–shell structure, and exhibited superparamagnetism, redox and pH stimuli-responsive features. The hybrid copolymer micromicelles were stable and nontoxic, and could entrap hydrophobic anticancer drug, which was in turn swiftly and effectively delivered from the drug-loaded micromicelles at special microenvironments such as acidic pH and high reactive oxygen species.

**Conclusion:**

This class of stimuli-responsive copolymer materials is expected to find wide applications in medical science and biology, etc., especially in drug delivery system.

**Electronic supplementary material:**

The online version of this article (doi:10.1186/s12951-017-0309-y) contains supplementary material, which is available to authorized users.

## Background

Stimuli-responsive block copolymer drug carriers are a class of functional nanoscaled drug delivery systems (DDS) [1–3]. As a drug release vehicle, they have attracted broad attention due to their unique properties and wide applications in the fields of biomedical nanotechnology [3–5]. This kind of drug carriers can promptly deliver drugs through alterations of the structure, conformation and configuration of carriers when they are stimulated by some special bioenvironments in vivo/in vitro, and physical or chemical factors such as light, temperature, pH, ultrasound, mechanical stress, reduction/oxidation, enzymes, ions, glucose, magnetic fields, solvent, voltage and electrochemistry [3, 4]. The stimuli-responsive DDS can reduce or avoid the non-controlled release of drugs and enhance the release efficiency of drugs in targeted areas. So far, the stimuli-responsive drug carrier materials sensitive to external environment conditions have become the hot spot of the study of targeted agents [1, 3, 4]. However, a majority of them just touch single-stimulus responsivity. Generally, stimulating factors in tumor and/or pathological microenvironments are not a result of a single stimulus, but a combination of environmental changes including pH, temperature, reducing substances, enzyme concentrations, reactive oxygen species (ROS) and adenosine-5′-triphosphate (ATP) [5]. Consequently, the single stimulus-responsive drug carriers cannot well respond to the complex functions and environments of living systems and easily suffered the problem of low release accuracy and some side effects [6, 7], which in turn cannot achieve optimal therapy efficiency and meet the demands for efficient overall therapy. Therefore, engineering and developing new drug carrier materials with dual or/and multiple stimuli responsiveness is considered to be a very important future direction.

Dual/multiple stimuli-responsive (polymer) materials as a new kind of ‘smart’ or ‘intelligent’ materials can produce significant changes in physicochemical properties upon triggered by tiny environmental stimuli [8–11]. Because of their unique properties, they are widely applied in the fields of drug delivery, diagnosis, tissue engineering, “smart” optical system, biological sensors, microelectromechanical system, coating and textiles, etc. In particular, dual/multi-stimuli responsive intelligent materials provide a good carrier platform for anticancer drug delivery by incorporating two or more stimuli responsive elements in DDS, exhibiting unique advantages and great development potentials. This gives us a unique opportunity to fine-tune their response to each stimulus independently, and augment the controlling modes of DDS and precisely modulate drug release profiles via the synergistic effect of different stimuli [6, 7]. Thus, formulation of new materials that can respond to specific changes of multiple stimuli is highly beneficial to achieve better drug controlled release profiles, better therapeutic effect and more systematic release kinetics. Dual-/multi-stimuli responsive smart materials include hydrogels, magnetic nanoparticles and/or microspheres, block copolymers and organic–inorganic hybrids, etc. [2–4, 6, 9]. Among them, redox, pH, temperature, enzyme, ATP, optical and magnetic responsive smart materials are especially intriguing and favored since more functions and finer modulations can be achieved. Dual/multi-stimuli responsive polymer materials can be obtained by introducing or combining various responsive moieties, namely, by combining monomer units having pH, temperature, electric, light and magnetic responses, etc. One has made many efforts to exploit dual or/and multiple stimuli responsive DDSs, for instance, temperature/pH, magnetic fields/pH, light/temperature, magnetic fields/temperature dual stimuli responsive DDS [4, 8], to accomplish more precise drug release in cancer microenvironments. Several researchers have done pioneering researches regarding dual- or/and multi-stimuli-responsive nanocarriers and made important achievements. Kang et al. [12] designed and fabricated a type of noncovalently connected copolymers by inclusion interaction for tunable release kinetics, which exhibited temperature and redox dual stimuli responsiveness. Li et al. [13] synthesized dual-stimuli sensitive keratin graft poly(*N*-(2-hydroxypropyl)-methacrylamide) sensitive to glutathione and trypsin, achieving the complete release of the payload. Wu et al. [14] developed a pH and thermo dual-controllable composite structure based on mesoporous silica nanoparticles encapsulated in a copolymer–lipid bilayer as a triggerable drug delivery carrier. Callahan et al. [15] designed triple stimulus-responsive polypeptide nanoparticles, enhancing intratumoral spatial distribution. Behzadi et al. [16] reported syntheses of triblock terpolymer and blends of diblock copolymers for nanocapsules that respond to oxidation and changes in pH and temperature. Priegue and Crisan et al. [17, 18] synthesized a versatile scaffold, poly(acryloyl hydrazide), which was in turn in situ functionalized for nucleotide and interfering RNA (siRNA) delivery; this work delineates a beautiful blueprint for developing and high-throughput screening and even future discovery of new functional polymeric materials with important biological applications. Dokania et al. [19] reported the formation of micromicelles, and examined the response to temperature and light, and the effect of chain length on the micelle forming properties, improving the oral absorption of the poorly-soluble drug. More recently, some recent studies and progress showcasing the construction and character of the multi-stimuli-responsive polymer materials including dual-, triple- and even quadruple-stimuli responsiveness were reported by Huang and Guragain et al. [20, 21]. These seminal studies motivate us to make further effort to provide valuable exploration and insights.

Since the heterogeneous structure and distribution of the tumor blood vessels may lead to the unique characteristics of the circulation inside the tumor, tumor cells exhibit various microenvironments, relatively high temperature (> 37 °C), low pH (5.8–7.1) and high content of ROS in comparison with normal cells [22, 23]. These extracellular tumor microenvironments provide strategies for increasing tumor selectivity and more effectively delivering drugs by a synergetic effect. Magnetic iron oxide nanoparticles (Fe_3_O_4_ NPs) have widely used in the field of biomedicine as theranostic agents and magnetic targeting reagents. Multimodal functionalities can be achieved by conjugating with a variety of targeting moieties on the surface of magnetic Fe_3_O_4_ NPs [24], and thus highly-desirable multiple stimuli-responsive materials can be developed based on Fe_3_O_4_ NPs.

In this context, our objective is to engineer and synthesize dual-stimuli-responsive hybrid Fe_3_O_4_ graft poly(acrylic acid)-*block*-poly(2-methacryloyloxyethyl ferrocenecarboxylate) block copolymers (Fe_3_O_4_-*g*-PAA-*b*-PMAEFC) to modulate physiochemical properties and drug release behavior of the assembled micromicelles. Albeit similar studies were conducted employing PAA and ferrocenium/ferrocene pairs for pH-/redox-responsive drug delivery [15, 16, 20, 21, 25, 26], this work possesses itself feature. The unique structure and dual stimuli-responsiveness make the micromicelles show obvious synergistic effect and adapt to the demands of various microenvironmental changes by simultaneously responding to multiple stimuli, thus maximizing the release amount of drugs at lesion or cancer locations and improving the bioavailability and targeting efficiency of drugs. This will be much necessary and significative for precisely switching on and off the release of the encapsulated guest drug molecules.

## Results and discussion

### Synthesis and characterization of the hybrid block copolymers

**Scheme 1:**
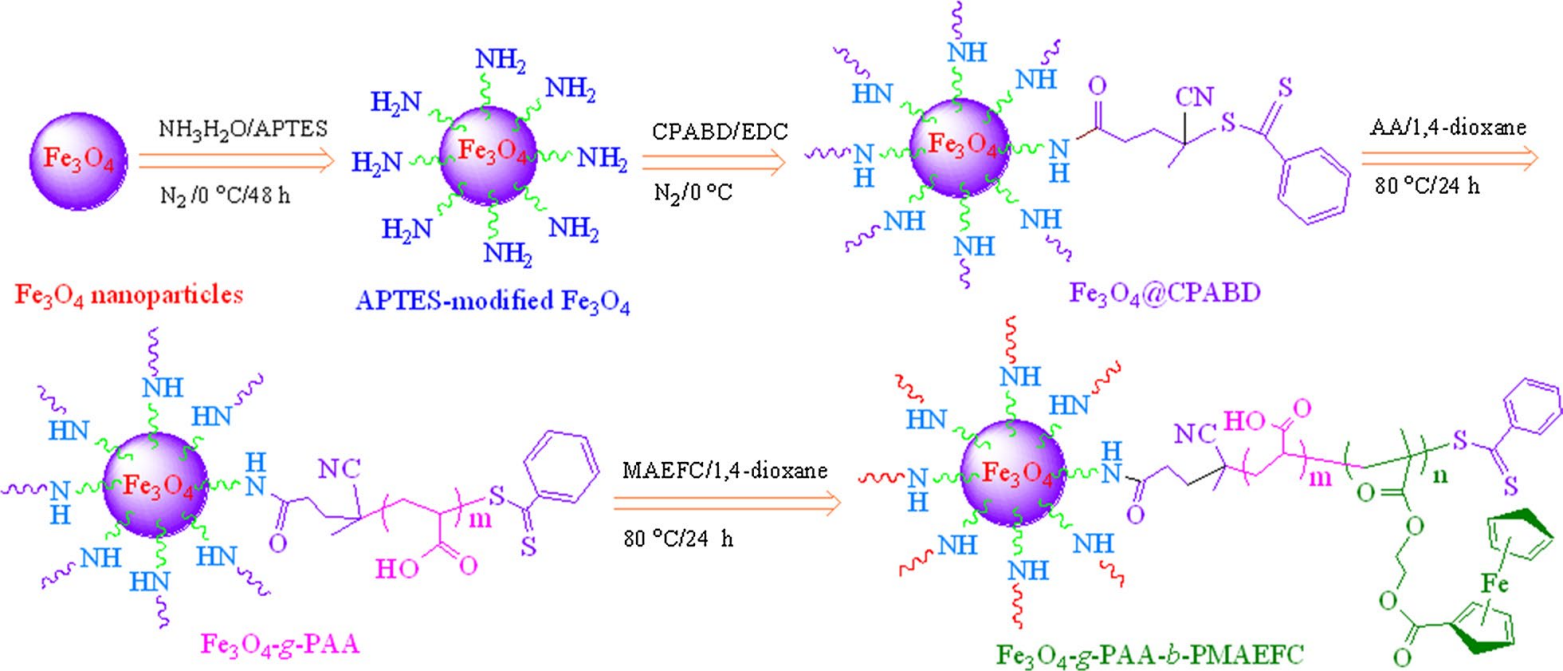
Representative synthesis scheme of Fe_3_O_4_-*g*-PAA-*b*-PMAEFC hybrid block copolymers

The synthesis of Fe_3_O_4_-*g*-PAA-*b*-PMAEFC hybrid block copolymers was conducted through a five-step strategy, as presented in Scheme 1. Magnetic Fe_3_O_4_ nanoparticles (Fe_3_O_4_ NPs) were first prepared by co-precipitating Fe^2+^/Fe^3+^ ions in an ammonia solution [27]. To conduct a surface reversible addition-fragmentation chain transfer (RAFT) reaction, Fe_3_O_4_ NPs was modified with (3-aminopropyl)triethoxysilane (APTES) [28]. The amino content on the surface of the modified Fe_3_O_4_ NPs was determined by potentiometric titration to be 18.25 mmol g^−1^; details were described in Additional file 1. This value is higher than that for the modified Fe_3_O_4_ NPs of the same size reported elsewhere [29]. Suppose that the APTES molecules are considered as a sphericity, and arrange on the surface of Fe_3_O_4_ NPs as per a monomolecular layer. Theoretically, there should be 484 APTES molecules covering on the surface of a Fe_3_O_4_ NP, viz., a surface coverage of 2.32 × 10^−10^ mol cm^−2^ (the calculation is described in Additional file 1) [30]. Actually, there are ca 4.23 APTES molecules covering on the surface of a Fe_3_O_4_ NP based on the amino contents of 18.25 mmol g^−1^. Consequently, the extent of the APTES particle coverage on the surface of Fe_3_O_4_ NPs is about 0.87%, or 2.03 × 10^−12^ mol cm^−2^. This value is significantly smaller than the saturated surface coverage of the theoretical monolayers on Fe_3_O_4_ surfaces mainly because the –OH groups on the surface of Fe_3_O_4_ NPs are less, and possess weak reaction capacity. Anyway, the amino modification of Fe_3_O_4_ NPs is conductive to the surface RAFT polymerization.

**Fig. 1:**
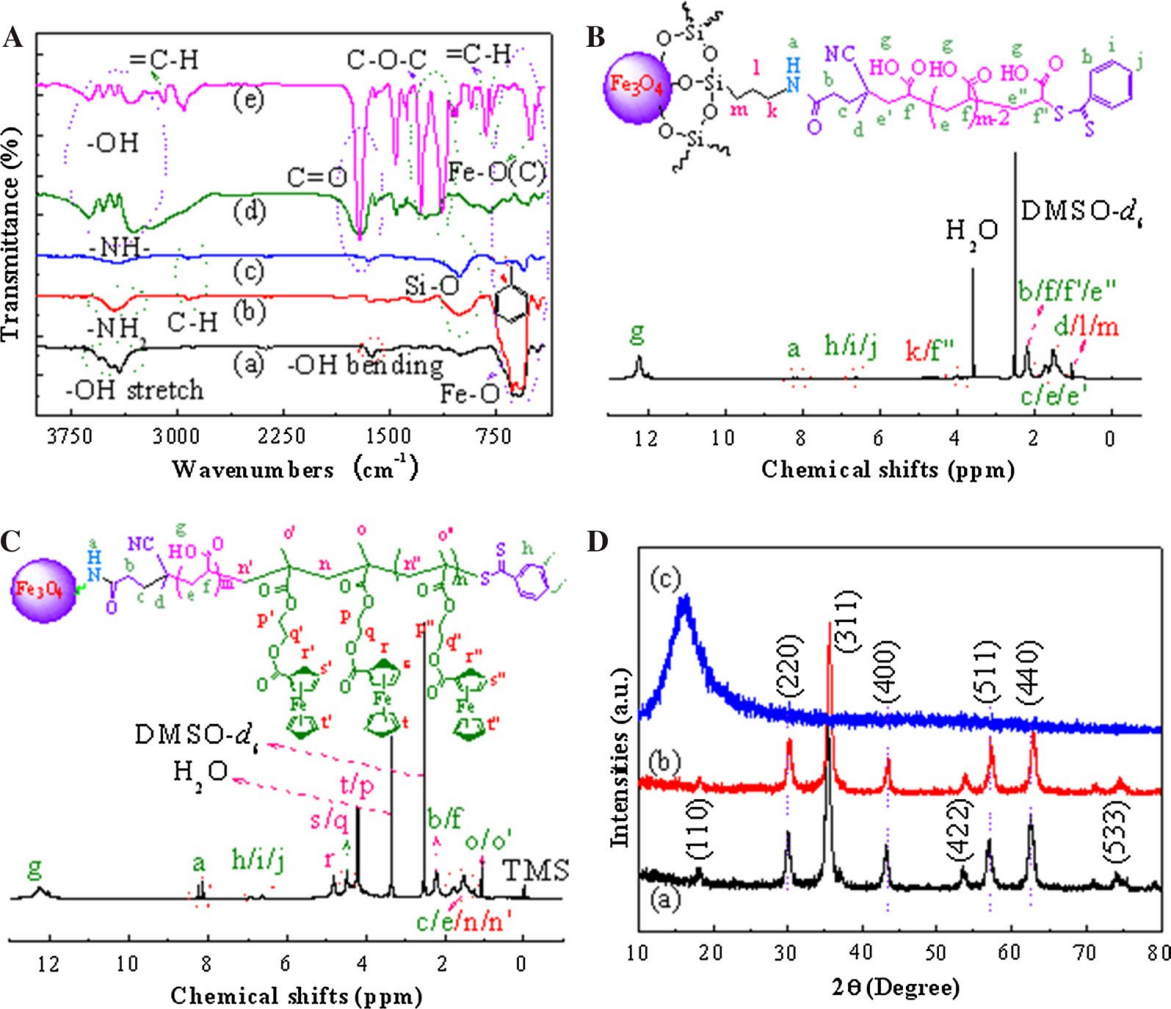
FTIR spectra (**A**) of (a) Fe_3_O_4_, (b) APTES-modified Fe_3_O_4_, (c) Fe_3_O_4_@CPABD, (d) Fe_3_O_4_-*g*-PAA and (e) Fe_3_O_4_-*g*-PAA-*b*-PMAEFC, and ^1^H NMR spectra of **B** Fe_3_O_4_-*g*-PMAA (DMSO-*d*_6_) and **C** Fe_3_O_4_-*g*-PAA-*b*-PMAEFC (DMSO-*d*_6_), and **D** XRD patterns of (a) Fe_3_O_4_, (b) APTES-modified Fe_3_O_4_ and (c) Fe_3_O_4_-*g*-PAA-*b*-PMAEFC at 25 °C using DMSO-*d*_6_ as solvents

FTIR was used to confirm the chemical structure of the prepared hybrid block copolymers, as demonstrated in Fig. 1A. Strong spectrographic vibration bands of Fe_3_O_4_ NPs emerge at 450, 587 and 630 cm^−1^ ascribed to the characteristic Fe–O bonds; weak and broad bands at 3415–3555 and 1620 cm^−1^ are assigned to the –OH stretch and bending modes of the free or absorbed water on the surface of ferrite nanoparticles, respectively [31, 32]. APTES-modified Fe_3_O_4_ NPs exhibit characteristic Fe–O vibrations at 450–630 cm^−1^ and additional vibration modes at 1006 and 2872–2926 cm^−1^ attributable to the Si–O and C–H stretch bands from APTES [33], respectively; peak at 3447 cm^−1^ is due to the –NH_2_ and residual –OH stretch. Fe_3_O_4_ graft 4-cyano-4(thiobenzoylthio)pentanoic acid (Fe_3_O_4_@CPABD) possesses characteristic FTIR vibration modes at 457–630, 690–742, 1006, 1635, 2855–2925 and 3440 cm^−1^ attributed to the Fe–O bonds, C–H out-of-plane bending of single (main) substituted benzene rings in chain transfer agents (CTAs), Si–O stretch, aromatic skeleton, C–H stretch and –NH_2_/–NH– stretch bands, respectively. In the case of Fe_3_O_4_ graft poly(acrylic acid) (Fe_3_O_4_-*g*-PAA) in Fig. 1A-d, the vibration bands reflecting poly(acrylic acid) (PAA) features appear at 3310–3620, 1709 and 1160–1245 cm^−1^ attributed to the associated and free –OH, C=O, and C–O stretch modes, respectively. The peak at 447–550 cm^−1^ is ascribed to the characteristic Fe–O bonds. These provide proof of existence of Fe_3_O_4_-*g*-PAA. FTIR spectra of Fe_3_O_4_-*g*-PAA-*b*-PMAEFC indicate presence of several new bands in Fig. 1A-e. The vibration bands at 3100 and 770–828, 1718, 1460, 1134–1276 and 495–543 cm^−1^ are attributed to the =C–H stretch and bending modes in cyclopentadienyl (Cp) rings, C=O, asymmetric C–C stretch of Cp rings, C–O–C, and asymmetric Fe–C or Cp-Fe stretch modes, respectively. The characteristic out-of-plane vibration bands of Cp rings occur at 1025, 1060 and 922 cm^−1^ [34]. These findings are in consistence with the FTIR spectra of 2-methacryloyloxyethyl ferrocenecarboxylate (MAEFC) in Additional file 1: Figure S1. Furthermore, we notice that a shoulder peak at 447 and the peak at 495–543 cm^−1^ are related to the Fe–O bonds in the bulk Fe_3_O_4_; a wide peak at 3330–3625 cm^−1^ is due to the associated and free –OH stretch in PAA moieties; the vibration peaks reflecting the C–H stretch and bending features at 2957 and 1376 cm^−1^ are more obvious probably due to the non-association in PMAEFC moieties.

^1^H NMR is a potent tool characterizing the chemical structure of polymers. As shown in Fig. 1B, the characteristic shift signals of PAA graft chains for Fe_3_O_4_-*g*-PAA appear at 12.22, 1.55–1.86 and 2.32 ppm predominately attributed to the carboxylic (–CO*OH*), methylene (–*CH*_*2*_CH(COOH)–) and methenyl (–CH_2_ *CH*(COOH)–) proton shifts, respectively. Several weak shift signals at 8.12–8.23, 6.60, 3.83–4.11 and 1.06 ppm are assigned to the shift characteristics of acylamino groups (–*NH*CO–), benzene rings (*C*_*6*_ *H*_*5*_–CS(=S)– in CTAs), methylene (–O–Si–CH_2_CH_2_ *CH*_*2*_NHCO–) and terminal methenyl (–CH_2_ *CH*(COOH)–(S=)SC–C_6_H_5_), and the ethylidene and methyl protons in CTAs and APTES residues (–O–Si–*CH*_*2*_ *CH*_*2*_CH_2_NHCO– and –CH_2_CH_2_C(CN)*CH*_*3*_–), respectively. ^1^H NMR spectra of Fe_3_O_4_-*g*-PAA-*b*-PMAEFC is depicted in Fig. 1C. The shift signal at 12.3 ppm is assigned to the proton feature of –COOH in PAA blocks, and other related signals with PAA moieties appear at 1.50–1.75 and 2.22 ppm corresponding to the methylene (–*CH*_*2*_CH(COOH)–) and methenyl (–CH_2_ *CH*(COOH)–) protons, respectively. The shift signals reflecting ferrocene structural features are located at δ = 4.81, 4.44 and 4.19 ppm; the proton shift signals of two methylene groups appear at δ = 4.21 and 4.33–4.40 ppm (*t*, –O–*CH*_*2*_ – *CH*_*2*_–O–), where the two signals at δ = 4.19 and 4.21 ppm, and 4.33–4.40 and 4.44 ppm are overlapped due to the ferrocene and methylene. Compared with MAEFC in Additional file 1: Figure S1, the slight shifts of methylene and methenyl proton signals to high fields are ascribed to the disappearance of double bonds and formation of the saturated C–C single bonds in the final product. The hydrogen proton resonance signals at 1.06 and 1.50–1.76 ppm are assigned to the methyl (–CH_2_–C(*CH*_*3*_)COO–) and methylene (–*CH*_*2*_–C(CH_3_)COO–) proton resonances in PMAEFC fragments, respectively.

The experimental molecular weight (MW) of the final copolymers was estimated by the peak area ratios of the –COOH peak in PAA blocks at 12.3 ppm to the peaks at 4.19–4.81 ppm related to ferrocene and methylene features; whilst the MW and chemical composition of PAA was obtained from the peak area ratios at 6.60 ppm (benzene rings) and 12.3 ppm (–COOH). Considering that the carboxylic acid proton integration is highly dependent on the protonation state as well as the formation of hydrogen bonds, the related calculations have been repeated and compared with the –CH– or –CH_2_ protons of the polymers. For the number-averaged molecular weight (Mn) of PAA blocks, the peak area ratio of benzene rings in CTAs at 6.60 ppm to the –CH_2_ protons at 1.55–1.86 ppm of the PAA polymers was calculated. It should be noted that albeit the –CH_2_ protons are overlapped with one –CH_2_ proton in CTAs, there is one –CH_2_ proton in PAA blocks shifting to 2.32 ppm, as shown in Fig. 1B. Moreover the –CH_2_ proton in CTAs can be negligible in comparison with the –CH_2_ proton in PAA repeating units. Likewise, during the calculation of Mn of PMAEFC blocks, the –CH protons of PAA blocks at 2.32 ppm can be used to calculate the integration area ratio by comparing the peaks at 4.19–4.81 ppm related to ferrocene moiety. The experimental results show that there is no significant differences between these Mn data through the peak area integration of different protons. The MW by ^1^H NMR is summarized in Table 1, which indicates that the Mn values match the feed ratios. It is derived from the Mn values by NMR that there are approximately 75, 39, 138 and 80 MAEFC repeating units, and 68 and 130 AA repeating units in copolymers. They are denominated P_1_, P_2_, P_3_ and P_4_, or Fe_3_O_4_-*g*-PAA_68_-*b*-PMAEFC_75_, Fe_3_O_4_-*g*-PAA_68_-*b*-PMAEFC_39_, Fe_3_O_4_-*g*-PAA_130_-*b*-PMAEFC_138_ and Fe_3_O_4_-*g*-PAA_130_-*b*-PMAEFC_80_, respectively. Size/steric exclusion chromatography (*SEC*) was used to characterize the MW and polydispersity index (PDI) of the resultant copolymers, as summarized in Table 1. These copolymers have increased MW with increasing the [monomer]/[initiator], which correspond to their high yields. Relatively low PDI values are given, but are still higher than those from atom transfer free radical polymerization (ATRP), which may lead to wide micelle size distribution.

**Table 1:** MW data of the prepared hybrid copolymer materials by NMR and *SEC*

Sample codes^a^	Formulation^b^	^1^H NMR M_*n*_	*SEC* data			*Yield*, %^c^
			M_*n*_	M_*w*_	*PDI*	
Fe_3_O_4_-*g*-PAA_68_-*b*-PMAEFC_75_, P_1_	1:75:100	30,560	49,670	74,010	1.49	78
Fe_3_O_4_-*g*-PAA_68_-*b*-PMAEFC_39_, P_2_	1:75:50	18,270	38,940	52,960	1.36	80
Fe_3_O_4_-*g*-PAA_130_-*b*-PMAEFC_138_, P_3_	1:150:200	56,580	68,750	96,940	1.41	78
Fe_3_O_4_-*g*-PAA_130_-*b*-PMAEFC_80_, P_4_	1:150:100	36,840	56,320	86,170	1.53	76

^a^The subscript figures represent degree of polymerization of PAA and PMAEFC blocks obtained from ^1^H NMR

^b^Molar ratios of Fe_3_O_4_@CPABD:AA:MAEFC

^c^Calculated by gravimetric method

Figure 1D depicts X-ray diffraction (XRD) patterns of Fe_3_O_4_, APTES-modified Fe_3_O_4_ and the resulting hybrid copolymers. Fe_3_O_4_ and APTES-modified Fe_3_O_4_ nanoparticles show characteristic XRD diffraction peaks at 2θ of 18.33°, 30.05°, 35.60°, 42.95°, 45.47°, 56.95°, 62.60° and 74.01°, which correspond to the lattice planes [110], [220], [311], [400], [422], [511], [440] and [533] [35]. This is well consistent with the standard XRD diffraction patterns of crystalline magnetites with regular octahedron cubic spinel structure (JCPDS Card No. 85-1436 or PDF#19-0629) [36]. The average crystal size (D) is calculated according to Scherrer formula:

1 $$ {\text{D}} = {{{\text{K}}\uplambda } \mathord{\left/ {\vphantom {{{\text{K}}\uplambda } {\left( {\upbeta { \cos }\uptheta } \right)}}} \right. \kern-0pt} {\left( {\upbeta { \cos }\uptheta } \right)}} $$

where *K* is Scherrer constant (0.89), λ is incident *X*-ray wavelength and equal to ca. 0.15418 nm, β is the peak full-width of half-maximum (rad), and θ is diffraction angel (°). The Fe_3_O_4_ and APTES-modified Fe_3_O_4_ NPs possess the mean sizes of 10.5 and 15.9 nm, respectively, which correspond to the [311] plane that shows strong diffractions. The XRD curve of the resulting hybrid copolymer gives a wide peak at 2θ of around 16.32°, which is attributed to the diffraction scattering of a large amount of amorphous copolymers encircling Fe_3_O_4_ NPs.

The thermostability of the hybrid copolymers are investigated by thermal gravimetric analysis (TGA), and representative TGA traces are shown in Additional file 1: Figure S2, indicating that Fe_3_O_4_-*g*-PAA-*b*-PMAEFC exhibits increased thermostability due to the π–π stacking of the ferrocenyl groups in the copolymers.

### Self-assembly micellization and physicochemical properties

**Scheme 2:**
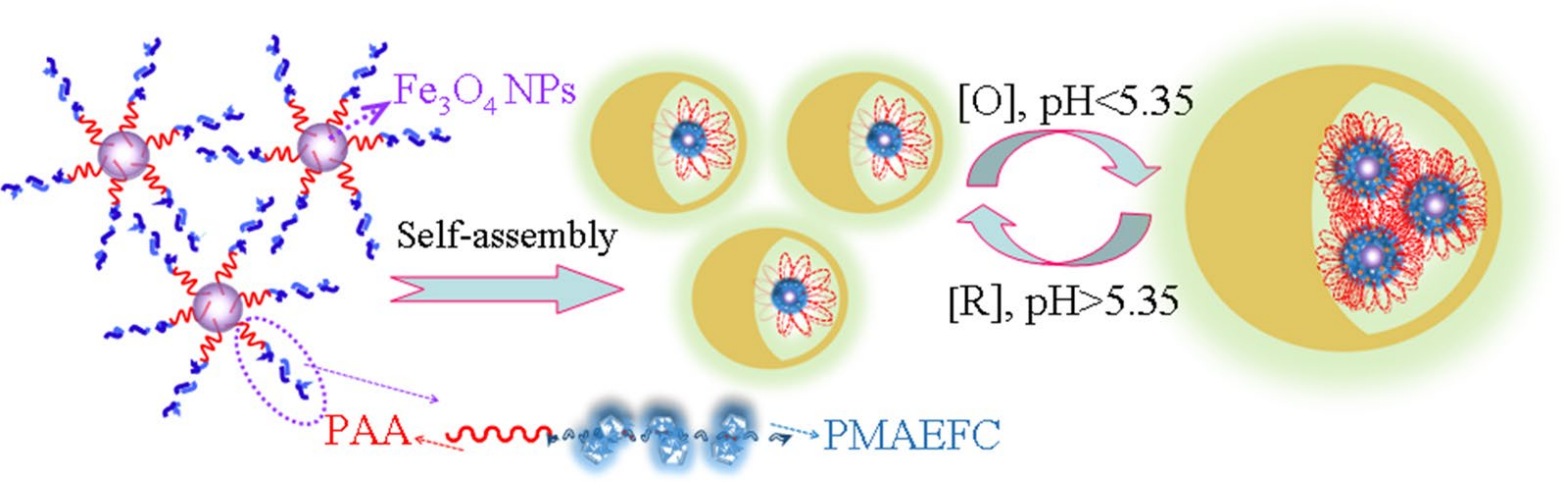
Diagrammatic drawing of reversible redox-responsive assembly and disassembly of typical block copolymer micelle aggregates

Fe_3_O_4_-*g*-PAA-*b*-PMAEFC is a hybrid amphiphilic block copolymer consisting of hydrophilic PAA chains and hydrophobic PMAEFC fragments with insoluble magnetic Fe_3_O_4_ NPs. Therefore, when they are put into aqueous solution, they are supposed to be able to spontaneously assemble into unique micelle aggregates with core–shell structure in a loop-type back-folding way, as illustrated in Scheme 2. This is because the hydrophilic PAA chains invariably incline to stretch to aqueous phase. The aggregation of these loop-like assembly micelles results in formation of the micromicelles with larger size. As an important physical parameter describing the formation of micelles, the critical micelle concentration (CMC) values are generally determined by fluorescent spectrometry using the fluorescence intensity ratios (*I*_3_/*I*_1_) of emission spectra of pyrene [37]. A polymeric concentration showing a discontinuous change in *I*_3_/*I*_1_ is defined as the CMC, as demonstrated in Additional file 1: Figure S3, and the estimated CMC values are summarized in Table 2. The CMC values increase with increasing hydrophilic AA structural units. Particularly, P_4_ bears a higher CMC value than P_1_ due to longer PAA chains for almost identical length of PMAEFC chains, which is consistent with the results reported elsewhere [38]. Dynamic light scattering (DLS) determination indicates that the micelles possess hydrodynamic diameter (D_*h*_) of about 190–260 nm in aqueous solution, forming a microscaled micelle aggregates (Table 2). The larger D_*h*_ values are correlated with more PAA-*b*-PMAEFC chains on the Fe_3_O_4_ surface.

**Table 2:** Physicochemical parameters of the hybrid copolymer micromicelles

Samples	CMC (mg ml^−1^)	*ξ* potentials (mV)^a^	D_*h*_ (nm)^a^	*PDI*
P_1_	0.167	− 80.35 ± 15.32	193 ± 28	0.225 ± 0.024
P_2_	0.305	− 108.25 ± 20.23	260 ± 15	0.147 ± 0.007
P_3_	0.295	− 133.05 ± 25.42	250 ± 39	0.276 ± 0.025
P_4_	0.346	− 128.25 ± 23.52	223 ± 45	0.286 ± 0.034

^a^The copolymer concentration is 1.0 mg ml^−1^ in deionized water

### Stimuli responsiveness of hybrid copolymer micromicelles

Considering the unique structure and composition of the hybrid copolymers consisting of Fe_3_O_4_ NPs, MAEFC units containing ferrocene groups and carboxylic acid units, they are anticipated to exhibit unique stimuli responsivities including magnetic, pH and redox responses.

**Fig. 2:**
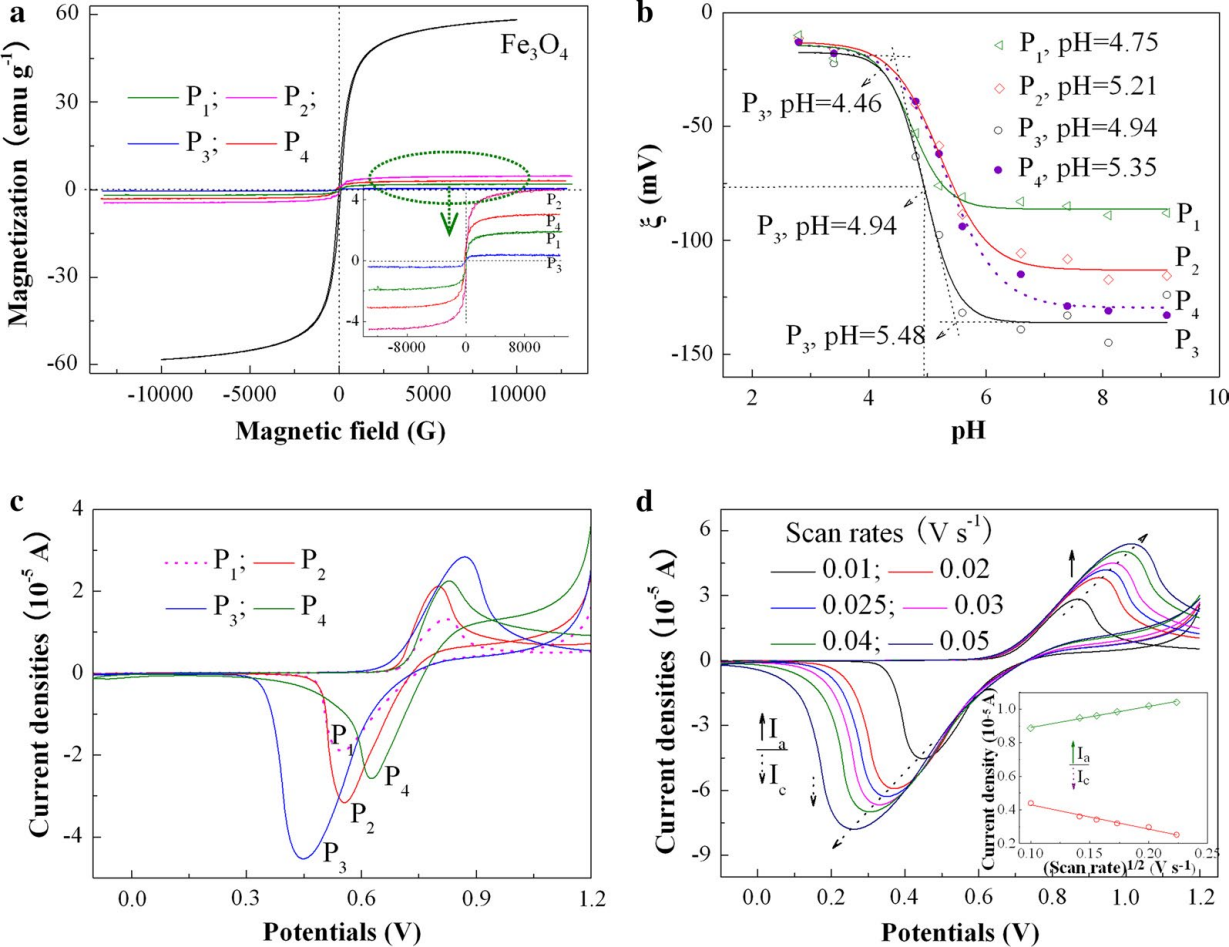
**a** Magnetization curves at 300 K of Fe_3_O_4_ NPs and P_1_, P_2_, P_3_ and P_4_ hybrid copolymers with various PAA and PMAEFC chain lengths; **b** change in *zeta* potentials of hybrid copolymers with pH; and **c** and **d** CV curves of **c** various hybrid copolymers at a scanning rate of 0.01 V s^−1^ and **d** typical P_3_ hybrid copolymer at different scan rates from 0.01 to 0.05 V s^−1^ in THF solution of 0.1 M (CH_3_CH_2_CH_2_CH_2_)_4_N(PF_6_) at 25 °C (the inset shows plots of peak currents *i*_p_ vs the square root υ^1/2^ of the scan rates). For **b**–**d** the concentration of the copolymer is 2 mg ml^−1^

Figure 2a shows the hysteresis loops of the hybrid copolymers at 300 K. It is seen that the hybrid copolymers exhibit ferromagnetism at different degrees in the presence of magnetic fields, with the saturation magnetization (*M*_s_) of about 1.95, 4.71, 0.42 and 3.08 emu g^−1^ corresponding to P_1_–P_4_ in sequence. This value is significantly lower than that of pure Fe_3_O_4_ NPs of ca 58.14 emu g^−1^ because of the grafting of a large amount of the copolymers with no magnetism on the surface of Fe_3_O_4_. The *M*_s_ values hinge on the MW or the length of PAA and PMAEFC chains, and the P_2_ and P_4_ have higher *M*_s_, which are consistent with their low MW in Table 1. Therefore, the hybrid copolymers with optimal magnetic properties can be obtained through tailor-making the length of the graft chains or/and modulating the amount of Fe_3_O_4_ NPs in micromicelles. When the external magnetic field is removed, the magnetism almost completely fades, with negligible coercivity (*H*_c_) less than 8.02 Oe (about 0.64 kA m^−1^) and rarely remanence (*M*_r_) smaller than 0.25 emu g^−1^, as summarized in Additional file 1: Table S1. This illustrates that the hybrid copolymer submicron particles are superparamagnetic and show characteristics of soft magnetic materials although the *M*_s_ values are significantly less than those of pristine Fe_3_O_4_ NPs [39]. This magnetic responsivity is anticipated to be potentially applied in magnetically targeted therapy of tumor.

On the other hand, PAA moieties as a weak acid have a p*K*a of 4.5–4.7 [40], and thus the alterations of pH would influence the protonation and deprotonation of carboxyl groups. The change in *zeta* potentials (ξ) of the hybrid copolymers with pH was measured to investigate pH response, as displayed in Fig. 2b. It is obviously noticed that the ξ values remain almost unchangeable below pH 4.46 (< p*K*a of PAA) due to the protonation of carboxyl groups and formation of hydrogen bonding interactions among –COOH. As pH increases (> p*K*a of PAA), the PAA moieties start to be partially ionized and the hydrogen bonds are gradually destroyed. As a consequence, the ξ absolute values abruptly increase. The deprotonation or disassociation of the carboxyl groups lead to formation of a lot of carboxylic anions, and thus a large number of the negative charges emerge on the micromicelle surfaces. When pH is above 6.03 for P_2_ or 5.48 for P_3_ (> p*K*a of PAA), the PAA chains are completely ionized and almost fully stretched, and the negative charges are full of the micelle surfaces. As a result, the ξ values reach at a maximum of about − 113.4 mV for P_2_ and − 135 mV for P_3_. These findings imply that the prepared micromicelles have preferable pH sensitivities. The pH phase transition points are defined as the pH value at which half of the total increase in *zeta* potentials occur, and are estimated to be about 5.21 for P_2_ and 4.94 for P_3_, slightly higher than the p*K*a of PAA moieties. The difference in ξ values and pH phase transition points between P_2_ and P_3_ is due to the grafting of more PAA chains for P_3_ on the surface of Fe_3_O_4_ NPs. The disassociation of hydrogen bonds and the ionization of more –COO^−^ groups are distributed at the outer shell layers of micelles, and thus P_3_ has higher ξ than P_2_. High ξ in pH of above 5.21 and/or 4.94 suggests the increased micromicelle stability in the simulated physiological environment. Likewise, P_1_ and P_4_ also exhibit pH responsivity, and the pH transition points are estimated to be approximately 4.75 and 5.35, respectively, as shown in Fig. 2b. The pH-induced responsivity can also be testified through micelle size change, viz. the D_*h*_ change determined by DLS, as tabulated in Table 3. For all the hybrid block copolymers, the D_*h*_ values at pH of 4.8 are larger than those at pH of 7.4, probably because of the inter-micelle aggregation caused by hydrogen bonds between the protonated –COOH groups in PBS of pH 4.8. This would lead to low *zeta* potentials, which are in consistent with the above discussion. In contrast, in PBS solution of pH 7.4, a large number of –COO^−^ anions are scattered on the small-size micelle surface based on Fe_3_O_4_ NPs with large specific area, leading to considerably high *zeta* potentials. The pH response provides a new choice of drug controlled release.

**Table 3:** Change in D_*h*_ values for the prepared copolymer micromicelles

Samples		P_1_	P_2_	P_3_	P_4_
D_*h*_, nm	pH 4.8	308 ± 40	503 ± 13	554 ± 20	406 ± 60
	pH 7.4	187 ± 22	252 ± 15	245 ± 39	218 ± 45
PDI	pH 4.8	0.253 ± 0.023	0.125 ± 0.005	0.224 ± 0.009	0.289 ± 0.036
	pH 7.4	0.212 ± 0.021	0.156 ± 0.008	0.254 ± 0.012	0.277 ± 0.045

The copolymer concentration is 1.0 mg ml^−1^

The electrochemical properties of the hybrid copolymers are investigated by cyclic voltammetry (CV), as demonstrated in Fig. 2c. Analyses reveal that the increase of the compositional ratios of MAEFC to AA leads to increased anodic oxidation potentials (E_p,anodic_), which can be inferred from the E_p,anodic_ comparison of P_1_ with P_2_, P_3_ with P_4_, and P_2_ with P_3_ (the E_p,anodic_ values are 0.813, 0.800, 0.871 and 0.829 V for P_1_, P_2_, P_3_ and P_4_, respectively). The shift of redox potentials is ascribed to the existence of ferrocenyl groups in targeted copolymers instead of the effect of solvents and diffusion coefficient [41]. Consequently, the hybrid copolymers with a large amount of ferrocenyl groups have high E_p,anodic_ values and are difficult to be oxidized. Nevertheless, the reversibility of the electrode process is increased with decreasing the ferrocene contents, and the peak separation (ΔE) for P_1_, P_2_, P_3_ and P_4_ is 0.264, 0.244, 0.425 and 0.203 V, respectively. P_2_ than P_1_, P_4_ than P_3_, and P_2_ than P_3_ have smaller ΔE; this is because the mass diffusion and the charge transfer between the active sites are more difficult to conduct in the case of high ferrocene contents [42]. To reveal the electrode process mechanism of the hybrid copolymer film, the CVs of typical P_3_ at different scan rates were determined in Fig. 2d. It is noticed that the reduction peaks shift cathodically and the oxidation peaks shift anodically as the scan rates increase, and thus the ∆E is augmented. Further investigation reveals that the ∆E values of the modified electrodes linearly increase with the scan rate (Additional file 1: Figure S4), suggesting that the electrode process is quasireversible [43]. It is also observed that as the scan rate increases, the redox peak currents of the modified electrodes increase, but the anodic current is smaller than the cathodic current. The peak currents (*I*_p_) has a direct proportion with the square root of the scan rate (ν^1/2^), giving a well-defined linear relationship between *I*_p_ and ν^1/2^ with high degree of fitting, *I*_p,c_ = − 1.4453ν^1/2^ + 0.5748 (R^2^ = 0.9761) and *I*_p,a_ = 1.2546ν^1/2^ + 0.7655 (R^2^ = 0.9945), as shown in inset of Fig. 2d. This indicates that the redox electrochemical process of the hybrid copolymers in solution is quasi-reversible diffusion-controlled [44].

**Fig. 3:**
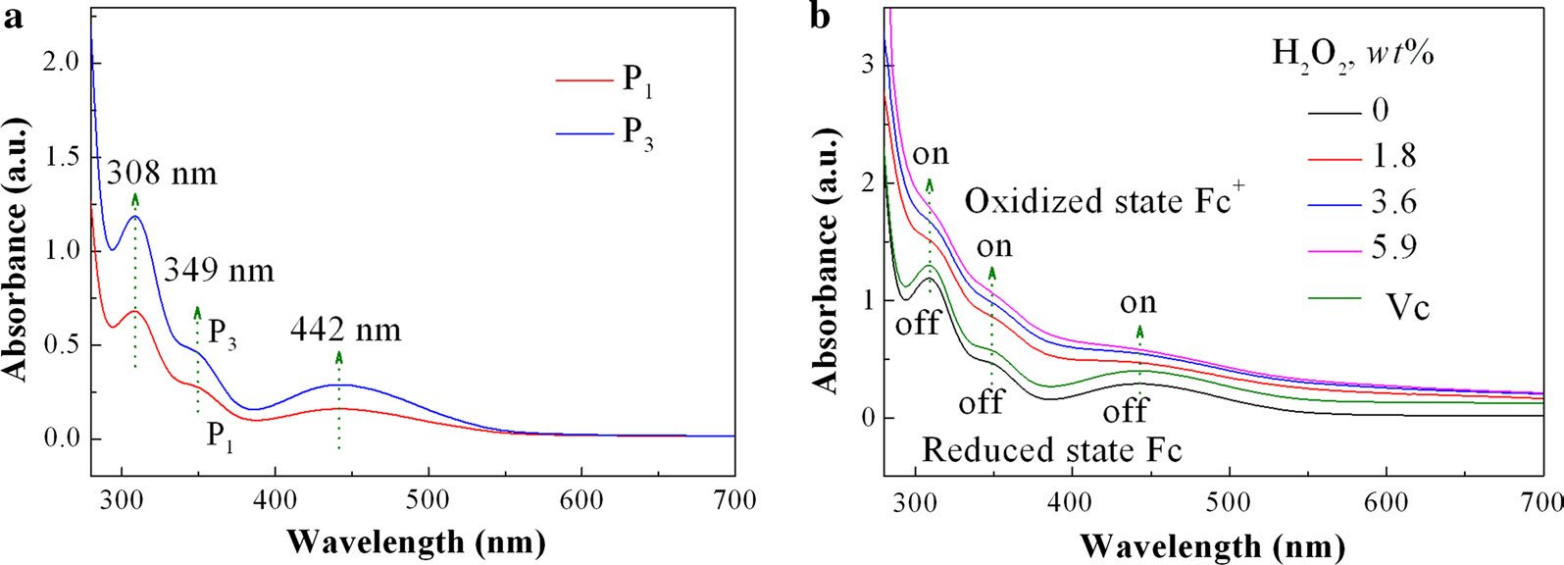
UV–vis spectra of **a** the prepared hybrid copolymer micelle aggregates with different hydrophilic/hydrophobic length ratios, and **b** typical P_3_ sample showing reversible redox transition between ferrocene and ferrocenium (concentrations: 0.25 mg ml^−1^) in DMF before and after H_2_O_2_ oxidation with concentrations (%) of 0.0, 1.8, 3.6 and 5.9, and then reduction by ascorbic acid (Vc)

Ultraviolet visible spectroscopy (UV–vis) was adopted to further explore the reversible redox stress responsiveness, as depicted in Fig. 3. Clearly, the characteristic peaks of the ferrocene moieties (Fc) in reduction state emerge at 442, 349 and 308 nm for the hybrid copolymers, and the peak intensities vary with the ferrocene contents or hydrophilic/hydrophobic length ratios, which is attributed to the special π–π conjugation structure of the Fc [26]. After the representative P_3_ is oxidized by hydrogen peroxide (H_2_O_2_), these electronic spectra disappear or wear off because of transition of the neutral Fc in reduced state to the ferrocenium cation moieties in oxidized state (Fc^+^) [26], depending on the concentration of H_2_O_2_. However, these characteristic electronic spectra reappear after Fc^+^ cations are reduced by ascorbic acid (Vc) ascribed to the transition of Fc^+^ to Fc. These results testify that the Fc–Fc^+^ and Fc^+^–Fc transition, or others, the oxidation and reduction process of P_3_, is completely reversible, producing reversible on–off switch behavior. Likewise, in the case of sodium hypochlorite (NaClO) and Vc as redox agents, P_3_ also shows reversible redox stress on–off responsiveness (Additional file 1: Figure S5).

**Fig. 4:**
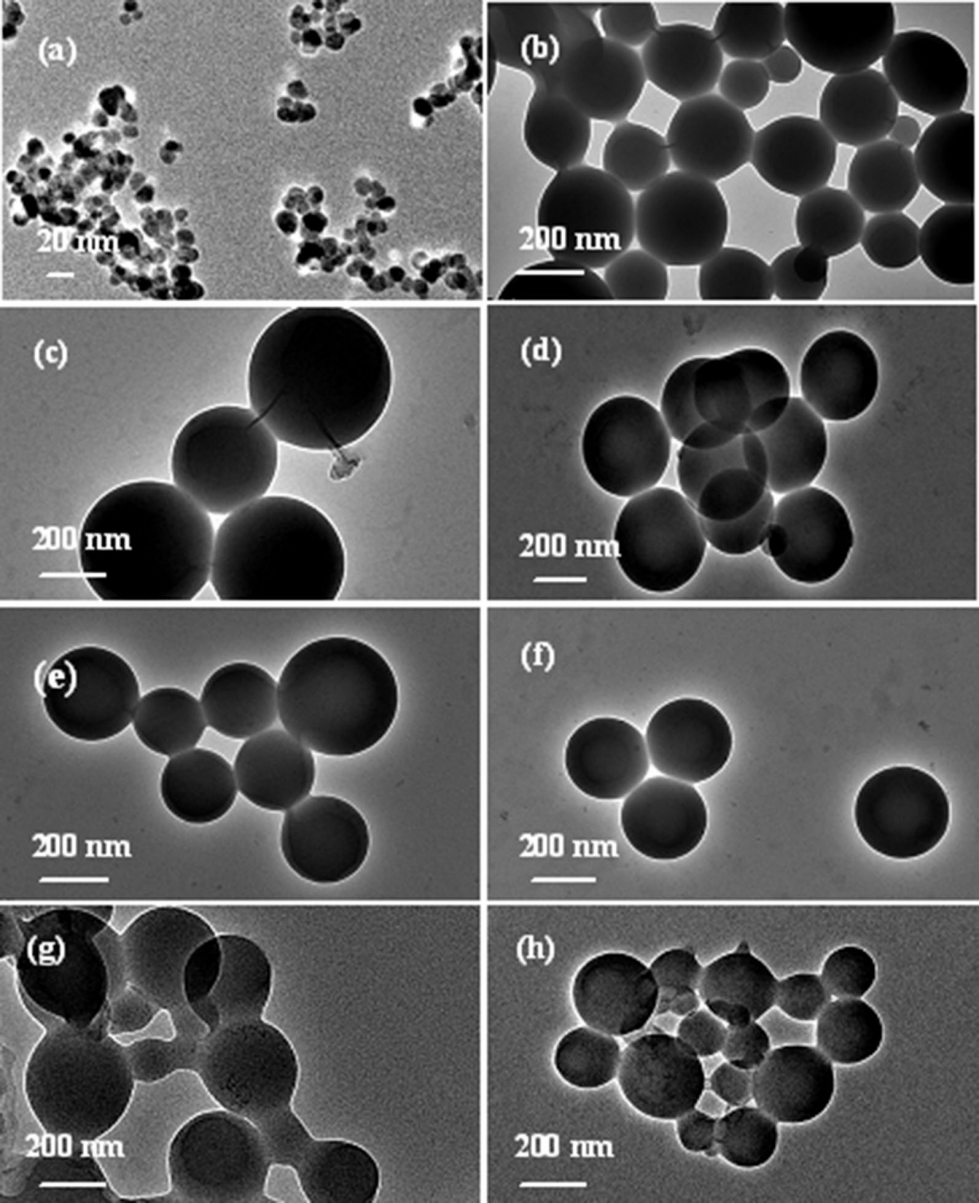
TEM microimages of **a** Fe_3_O_4_ NPs and **b**–**e** representative P_3_ hybrid copolymer micromicelles at various redox states (polymer concentration is 0.5 mg ml^−1^): **b** the original micelles, the micelles oxidized by **c** H_2_O_2_ and **d** NaClO, the micelles reduced by Vc from the oxidized micelles by **e** H_2_O_2_ and **f** NaClO, and **g**, **h** the micelles at pH of 4.8 and 7.4, respectively

Transmission electron microscope (TEM) observations were carried out to obtain morphologies of the micelle aggregates at different redox states and pH values, and further provide insights into the nature of the redox species and pH-responsive moieties. Figure 4 displays TEM microimages of representative P_3_ micromicelles at various redox states and pH values. Fe_3_O_4_ NPs assume spherical morphologies with a mean particle size of about 11 nm, which is consistent with the crystal size by XRD. P_3_ takes on a well-defined globular core–shell micromicelle topologies at various redox states. The freshly-prepared P_3_ possess a relatively wide particle size range from 160 to 400 nm, with the mean size of ca 266 nm in Fig. 4b. The wide size distribution may be related to wide MW distribution in Table 1. In comparison, the oxidized micromicelles by H_2_O_2_ and NaClO exhibit larger particle sizes and narrower size distribution, with the average size of about 420 ± 30 and 360 ± 60 nm, respectively (Fig. 4c, d). This is because the neutral Cp_2_Fe groups switch to the Cp_2_Fe^+^ cations, leading to increase in hydrophilicity and swelling of the ferrocene moieties in cores [45]. The electrostatic repulsion among the charged particles also makes the micelles expand, and thus the particle size increases [46]. DLS measurements corroborate the change of the micelle size induced by oxidation. The *D*_h_ of P_2_ apparently increases from about 260 to 390–545 nm (Additional file 1: Table S2). Since H_2_O_2_ is a neutral oxidant molecule, it can effectively oxidize the hydrophobic ferrocenyl groups into the hydrophilic ferrocenium cations, enhancing the hydrophilicity and electrostatic repulsion of the micelle cores and swelling of PMAEFC domains [45, 47]. In contrast, NaClO is a salt of strong alkali weak acid, and exhibits strong oxidization in alkali media. As a result, ClO^−^ anions only allow selective or partial oxidization, resulting in locally swollen Cp_2_Fe^+^ domains [47]. Moreover, the electrostatic attraction between ClO^−^ or/and its product Cl^1−^ anions and the Cp_2_Fe^+^ domains at oxidization states impairs the electrostatic repulsion of the micelle cores. This leads to larger expansion and particle size for H_2_O_2_ than NaClO. After Vc is added, the Cp_2_Fe^+^ cation moieties at oxidized states are reduced to the neutral Cp_2_Fe groups. As a result, the hydrophilicity and electrostatic repulsion of the cores decrease or disappear, and then the particle size reduces to about 260 ± 70 and 260 ± 30 nm for the samples oxidized by H_2_O_2_ (Fig. 4e) and NaClO (Fig. 4f), respectively, nearly close to the original micelle size. The *D*_h_ for P_2_ also somewhat decreases (Additional file 1: Table S2). These findings further verify that the micromicelles possess good redox reversibility. The morphologies and sizes of the copolymer micromicelles are also influenced by the change in pH values, as shown in Fig. 4g, h. In comparison with that in aqueous solution, although the hybrid copolymers remain spherical topologies in the two pH media, the particle size differs from each other. At a pH of 4.8, the micellar interfaces become conjoint, and the particle size is in the range from about 260 to 345 nm with mean size 300 nm, higher than that of 266 nm in aqueous solution. This is ascribed to the protonation of carboxylic groups, formation of stronger hydrogen bonds and the hydrogen-bond and hydrophobic aggregation of the micromicelles in the medium of pH 4.8. At pH of 7.4, the sufficient ionization leads to the dissociation of hydrogen-bonding interactions and the deaggregation of the micromicelles, and the size of the micromicelles decreases to about 160–235 nm, with a mean size of about 200 nm. The TEM images of the micromicelle particles at the two different pHs reveal their pH responsiveness, which is in consistent with the conclusion drawn from DLS and *zeta* potentials measurements.

**Fig. 5:**
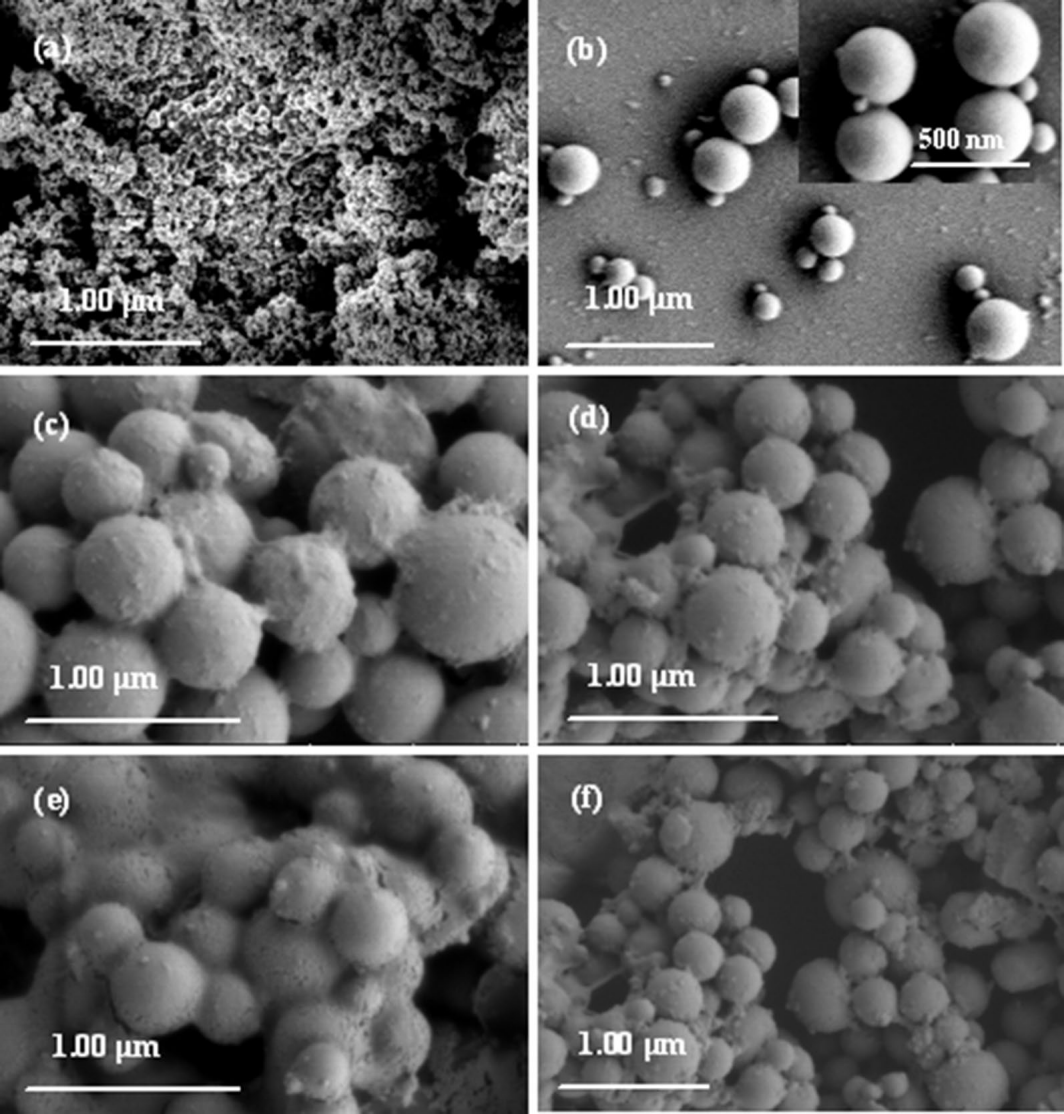
FESEM microphotos of **a** Fe_3_O_4_ nanoparticles and **b**–**f** P_3_ hybrid copolymer micromicelles at various redox states: **b** original micelles, **c** micelles oxidized by H_2_O_2_, **d** micelles oxidized by NaClO, **e** micelles oxidized by H_2_O_2_ and then reduced by Vc, and **f** micelles oxidized by NaClO and then reduced by Vc

Field emission scanning electron microscope (FESEM) was also used to intuitively observe the microtopography of the micromicelles at various redox states, as shown in Fig. 5. Fe_3_O_4_ NPs possess globular morphologies piled together, with uniform size distribution and a mean size about 13 nm, which is in agreement with XRD and TEM results. The original micromicelle particles take on smooth globular topologies, with a wide particle size distribution ranging from 150 to 370 nm, and most of them about 340–350 nm (Fig. 5b and its inset). After they are treated with H_2_O_2_ (Fig. 5c), remarkable irregular outgrowths emerge on the surface of the oxidized micromicelles. The size of most of the micromicelles expands to a mean diameter of ca. 520 nm ranging from 480 to 700 nm. However, the particle size distribution is more homogeneous. The reason is that the formation of hydrophilic ferrocenium cations (Fc^+^) in PMAEFC domains enhances swelling and electrostatic repulsion of the cores, as stated before. NaClO oxidization has similar phenomenon: the surface of the oxidized micromicelles become coarse and the micellar size is increased up to about 375 nm ranging from 250 to 450 nm (Fig. 5d). The morphologies of the Vc-reduced micromicelles have no significant difference from the oxidized ones, but the particle size decreases to about 250–450 and 215–570 nm, and the average size is about 350 (Fig. 5e) and 357 (Fig. 5f) nm, respectively, close to the original micromicellar size and the size distribution. It is concluded that the micromicelles possess fine reversible redox stress responsiveness or on–off switch properties.

Since TEM and SEM present the limited number of particles, while DLS gives wide size distribution before oxidization, it is necessary to conduct XPS measurements to provide insights into the nature of the redox species. Additional file 1: Figure S6 shows the high-resolution XPS spectra of Fe 2p. Before oxidation, the Fe 2p peaks are deconvoluted into a spin–orbit-coupled doublet with the binding energy (BE) of about 704.9 and 717.7 eV attributed to electrons from the Fe 2p_3/2_ and Fe 2p_1/2_ electronic levels, respectively. This signifies the existence of Fe^2+^ cations in the ferrocene-containing moieties, and no Fe^3+^ cation is detected. Thus, the vinylferrocene moieties are verified to remain stable upon preparation of the micromicelles. Only after oxidation, the BE of the above spin–orbit-coupled doublet is shifted into 705.7 and 718.5 eV, respectively, and a new peak emerges at around 709.2 eV, indicative of the presence of Fe^3+^ cations [48]. These findings provide the support for the interpretation of TEM, SEM and DLS results.

### Drug entrapment and dual-stimuli responsive drug release

Paclitaxel (PTX) is a kind of common hydrophobic anticancer drugs, and can be entrapped in the core of the micromicelles during their self-assembly. To investigate effect of copolymer compositions on the loading capacity and encapsulation efficiency, the loading capacity (LC) and the encapsulation efficiency (EE) of the PTX-loaded copolymer micromicelle drug preparations are determined, and the results are tabulated in Additional file 1: Table S3. It is clearly noticed that the LC and EE values are increased with enhancing the length of hydrophilic PMAA and hydrophobic PMAEFC chains probably due to longer chains can entrap more drug molecules and keep the micromicelles stable. As the length of PAA blocks remain unchangeable at 68 or 130 units, the copolymer micromicelles with longer PMAEFC chains can capture more PTX molecules, and thus offer relatively high LC and EE values. The possibility is that a subtle hydrophilic/hydrophobic balance can keep the encapsulated drug stable without precipitation, and accordingly an optimal drug formulation with relatively high LC and EE values can be achieved by varying block composition of copolymers.

**Fig. 6:**
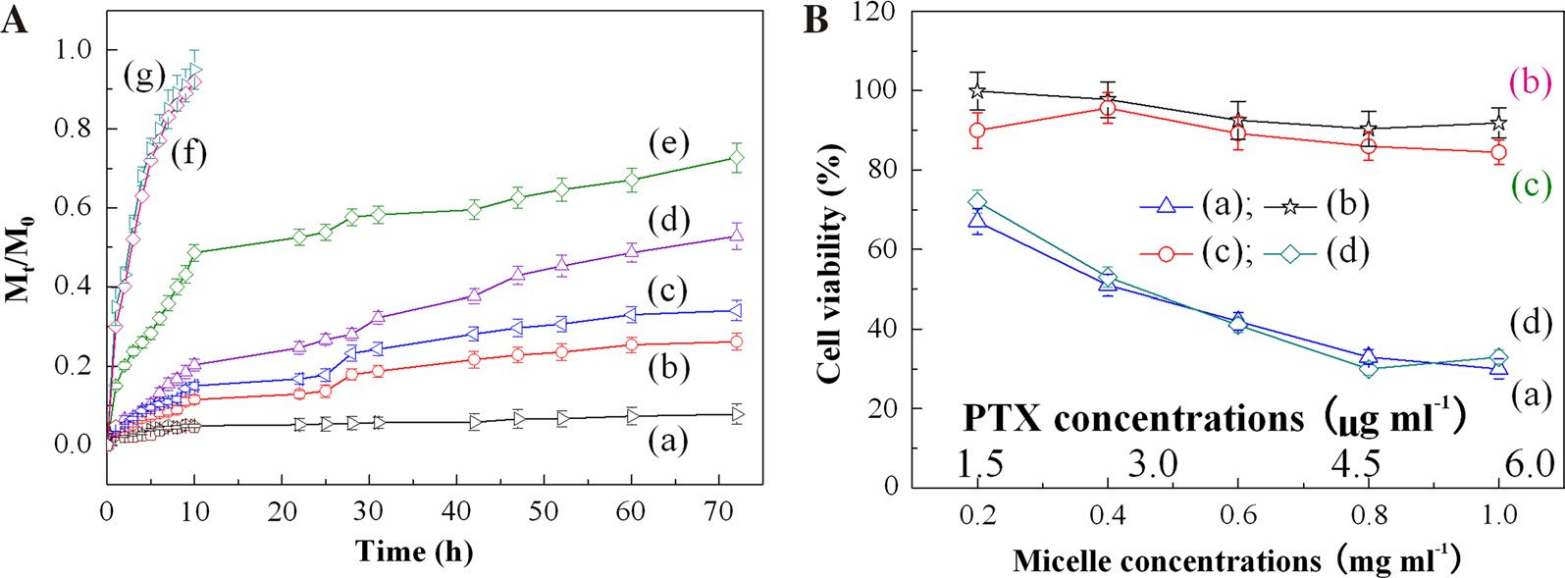
**A** Oxidization stress and pH triggered PTX drug release profiles from PTX-loaded P_4_ copolymer micromicelles at 37 °C and various pH and H_2_O_2_ concentrations: (a) pH 7.4, (b) pH 6.3, (c) pH 5.3, (d) pH 5.3 and 0.2% H_2_O_2_, and (e) pH 5.3 and 0.8% H_2_O_2_, and free PTX release at medium of (f) pH of 7.4 and (g) pH of 5.3 and H_2_O_2_ of 0.8%; and **B** cytotoxicity of (a) free PTX, (b) blank P_4_ and (c) PTX-loaded P_4_ micromicelles at pH 7.4, and (d) PTX-loaded P_4_ micromicelles at pH of 5.3 and a H_2_O_2_ concentration of 0.8% against A549 lung cancer cell lines at 37 °C after 24 h incubation

To verify the practical applications of the designed micromicelles as drug release carriers, PTX drug release profiles are investigated at various pH microenvironments and ROS prevailing in cancer cells, H_2_O_2_ with various concentrations, as shown in Fig. 6A. Interestingly, the PTX-loaded micromicelles exhibit remarkable oxidization stress and pH responsive drug release, as expected. As illustrated in Fig. 6A(a–c), at pH 7.4, the drug release rate is slow, and only about 7.8% PTX delivers from the PTX-loaded micromicelles after 72 h. However, as the pH decreases, PTX release rates are accelerated, and lower pH leads to more PTX release. The release amount of PTX at pH 5.3 reaches up to 34.1%, higher than that of 26.2% at pH 6.3. The results indicate an obvious pH-dependent PTX drug release. Even so, the release amount is still low for more effective cancer therapy. As H_2_O_2_ is adopted to induce PTX release, the PTX release rate is significantly enhanced, and the release amount increases with increasing the concentration of H_2_O_2_, as shown in Fig. 6A(c–e). After 72 h, the PTX release amount reaches 52.8 and 72.7% for 0.2 and 0.8% H_2_O_2_, respectively, higher than that of 34.1% without ROS triggering. In contrast, free PTX quickly deliver regardless of in normal physiological conditions (pH 7.4) or in high-concentration ROS H_2_O_2_ and acidic pH microenvironments (pH of 5.3 and H_2_O_2_ of 0.8%), producing burst release behavior. The cumulative release amount of PTX reaches more than 90% within 10 h, with no targeting controlled release observed, as depicted in Fig. 6A(f, g). Therefore, it is of great significance to develop the dual-stimuli responsive micromicelles as controlled and targeted drug release carriers. In this way, the PTX-loaded drug preparation can be quickly and accurately guided to the cancer sites, and fleetly deliver PTX at cancer cells through high-concentration ROS H_2_O_2_ and acidic pH microenvironments in cancer sites [22, 23, 49]. Meanwhile, the harm to normal cells or tissues can be maximally avoided because of the dual effects including pH and ROS species.

### Evaluation of the in vitro cytotoxicities

Non-toxicity or low cytotoxicity is highly desired for drug or/and gene release carriers, and evaluated by 3-[4,5-dimethylthiazol-2-yl]-2,5-diphenyltetrazolium bromide **(**MTT) assays with A549 lung cancer cell lines, as shown in Fig. 6B. The blank nanomicelles generate more than 90% cell viability even at a high concentration of 1.0 mg ml^−1^ (*p* < 0.05), showing almost nontoxic. The cell proliferation of the PTX-encapsulated micromicelles is, as expected, slightly suppressed compared with the blank counterpart due to a little amount of the in vitro drug release at pH 7.4. However, there are still more than 85% cell survival, exhibiting low cytotoxicity; this will not do harm to normal cells. In contrast, for free PTX only 67–30% cells survive in a dose-dependent way; this will kill cancer and normal cells simultaneously. MTT assays suggest that the entrapment of PTX in the copolymer micromicelles can effectively avoid the toxic and side effects from PTX during cancer therapy. Albeit MTT assay is generally evaluated at pH 7.4, the anticancer activity is evaluated under the condition of pH 5.3 and H_2_O_2_ concentration of 0.8% in consideration of the redox and pH dual-stimuli responsiveness of the prepared micromicelles, as well as the acidic environments and high ROS concentrations at tumour/cancer zones, as illustrated in Fig. 6B. Clearly, during the culture and circulation of the PTX-loaded micromicelles in simulated cancer zones, acidic microenvironments and high ROS concentrations trigger the release of a large amount of PTX from the PTX-loaded micromicelles, leading to a remarkable decline of the cell survival, with the half maximal inhibitory concentration (*IC*_50_) of about 3.38 μg ml^−1^, and thus an increase in anticancer activities and the therapy efficacy of PTX against cancer cells. This toxic effect is equivalent to that of free PTX (*IC*_50_ of ca 3.15 μg ml^−1^), thus significantly inhibiting the A549 lung cancer cell lines from the growth and spread, and showing a targeting therapy effect. The developed copolymer micromicelle can therefore be used to treat cancers as a potential drug controlled delivery carrier and effectively avoid the harm or damage to normal cells.

### Physical stability of the micromicelles with and without PTX

**Fig. 7:**
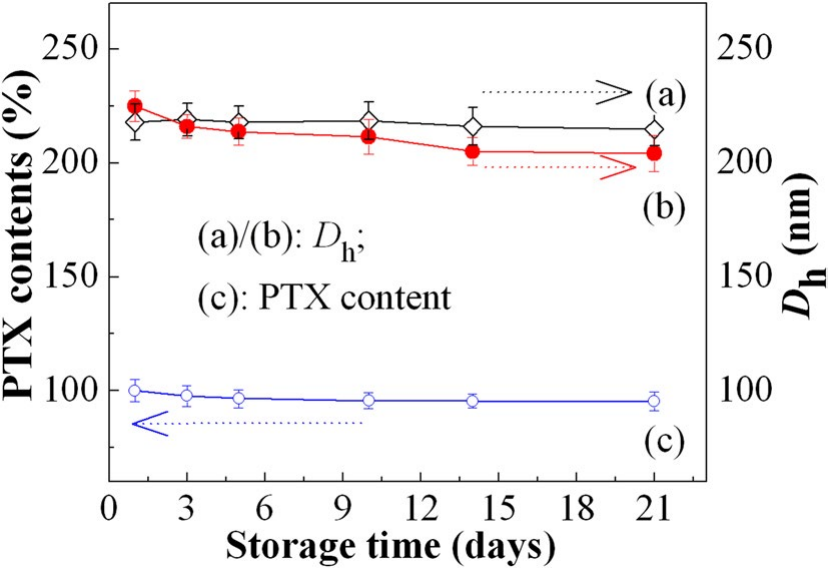
Change in D_*h*_ of (a) the blank and (b) the PTX-loaded micromicelles, and (c) PTX contents of the PTX-loaded P_4_ micromicelles in PBS of pH 7.4 at 4 °C during a 21-day storage

The physical stability of the (micro)micelles is crucial for their biological applications as DDS in that stable micelles can withstand dissociation and premature release of its cargo or payload after entry into the bloodstream [50]. The stability depends upon glass transition temperature, CMC, *zeta* potentials, drug loading as well as the interactions between the drug and the core-forming block (i.e., drug-core interactions) [50, 51]. In this study, we compare the dynamic stability of the blank micromicelle with that of the PTX-loaded micromicelle by determining change in D_*h*_ of the P_4_ micromicelle and the PTX content during a storage period of 21 days at 4 °C and pH of 7.4, and the results are shown in Fig. 7. Clearly, the blank P_4_ micromicelle possesses significantly high dynamic stability, and its *D*_h_ values remain almost unchangeable within experimental errors. The micromicelles always take on homogeneous colloidal dispersion, with no disassembly and aggregation or precipitation of the micromicelles observed. Although the D_*h*_ value of the PTX-loaded micromicelle declines from 224.7 to 210.0 nm after a 21-day storage, there is only about 6.5% change in size (Fig. 7b), slightly higher than that of the blank one, due to the loss or escape of a little amount of PTX from the hydrophobic core of the copolymer micromicelles. This loss or leakage can be monitored by gauging the change of PTX contents in micelle-based drug formulations, as shown in Fig. 7c. About a 4.8% PTX loss after 21 days is probably ascribed to the partial decomposition or slow delivery of PTX from the micromicelles, and no PTX aggregation or precipitation is observed, suggesting that the PTX-loaded P_4_ micromicelle remains good physical stability. Consequently, the copolymer micromicelles are applicable as potential drug targeted release carriers.

## Conclusions

In summary, dual-stimuli responsive Fe_3_O_4_-*g*-PAA-*b*-PMAEFC hybrid magnetic graft block copolymers with redox and pH responsiveness have successfully prepared through sequential RAFT techniques, as revealed by FTIR, ^1^H NMR, *SEC*, TGA and XRD. TEM and SEM observations disclose that the hybrid copolymers can spontaneously assemble and form globular core–shell micelle aggregates. *Zeta* potentials, VSM, CV, UV–vis, XPS, TEM, SEM and DLS measurements indicate that the hybrid copolymer micelles are in micron level, and exhibit unique pH, magnetic and quasireversible redox stimuli responsiveness that relies on the compositional ratios or the chain length of the blocks, with pH phase transition points of about 4.75–5.35. The dual-stimuli responsive micromicelles are stable, and low-toxic even at a high concentration of 1.0 mg ml^−1^. They can selectively and effectively deliver PTX at cancer/tumour tissues with low pH (4.5–7.2) and high ROS, whereas the premature leakage of PTX can be eliminated or minimized during the circulation in vivo (pH 7.4, low ROS), achieving an enhanced targeted therapy efficacy against cancer cells.

## Materials and methods

### Materials

Ferrocene carboxylic acid [(FCA, 98%, Tokyo Chemical Industry **(**TCI), Japan] and hydroxyethyl methacrylate (HEMA, 96%, Aladdin Industrial Corp., Shanghai, China) was purified by passing through neutral alumina column to remove the antioxidant and inhibitor prior to use [51, 52]. *N*,*N*′-Dicyclohexylcarbodiimide (DCC, 99%, Aldrich), dimethylaminopyridine (DMAP, 99%, Aldrich) and acrylic acid (AA, 99%, Macklin) were directly used without purification. 2,2′-Azobis(isobutyronitrile) (AIBN, 98%, Aldrich) was recrystallized from anhydrous ethanol. 4-Cyano-4(thiobenzoylthio)pentanoic acid (CPADB, 97%), a RAFT chain transfer agent (CTA), was supplied by the Strem Chemicals, Inc., USA. 1-Hydroxybenzotriazole (HOBT, 97%) was used as a protective agent of amido bonds [53, 54] and purchased from the Shanghai Macklin Biochemical Co., Ltd., China. 1-Ethyl-3-(3-dimethylaminopropyl)carbodiimide hydrochloride (EDC·HCl, 98%) as dehydrant was provided by the Aladdin Industrial Corp., Shanghai, China. (3-Aminopropyl)triethoxysilane (APTES, 99%) was purchased from the Aladdin Industrial Corp., Shanghai, China. Dichloromethane (DCM, 99.5%) and 1,4-dioxane (DIOX, 99.5%) were dried with CaH_2_ overnight and distilled under reduced pressure. Tetra-*n*-butylammonium hexafluorophosphate (C_16_H_36_F_6_NP, 98%) and acetonitrile (C_2_H_3_N, 99.8%) were purchased from Shanghai Darui Finechemical Co., Ltd., China.

### Methods

Magnetic Fe_3_O_4_ NPs were first prepared and modified with APTES as per references [27, 28], and the details were described in Additional file 1. To obtain RAFT CTAs, CPADB was introduced onto the surface of Fe_3_O_4_ NPs through esterification reaction [53, 54], and the product was labeled as Fe_3_O_4_@CPADB. Typically, APTES-modified Fe_3_O_4_ (0.0105 g, 0.1916 mmol) was dispersed in desiccative DCM (50 ml) under ultrasonication for 30 min. Then, CPADB (0.1014 g, 0.3634 mmol) and HOBT (0.0394 g, 0.2915 mmol) was added to the Fe_3_O_4_ dispersion solution with mechanical stirring. The mixture solution was cooled into 0 °C, and then EDC·HCl (0.1608 g, 0.8388 mmol) dissolved in desiccative DCM (10 ml) was dropwise added into the reaction vessel within 20 min with a constant pressure funnel under protection of N_2_. After the reaction proceeded for about 48 h at room temperature, the reaction mixture was separated by centrifugation and washed repeatedly by ethyl alcohol and deionized water until the solution indicated neutrality. The precipitates were dried in a vacuum oven at 40 °C, offering final product Fe_3_O_4_@CPADB.

Synthesis of Fe_3_O_4_-*g*-PAA was performed through a RAFT polymerization avenue at molar ratios of Fe_3_O_4_@CPADB/AA/AIBN of 1:75:0.25. Typically, in a 25 ml Schlenk flask, AA (0.986 ml, 14.3718 mmol), Fe_3_O_4_@CPADB (0.0131 g, 0.1920 mmol), and AIBN (0.0078 g, 0.0479 mmol) were dissolved in 1,4-dioxane of 8 ml. The mixed solution was degassed by a successive freeze–pump–thaw cycle three times and ultimately suffused with N_2_. The reaction system was heated to 80 °C, and the reflux reaction was performed for 24 h with violent magnetic stirring. The crude product was repeatedly precipitated in anhydrous ether five times to remove excess reactants. The resultant Fe_3_O_4_-*g*-PAA precipitates were dried in a lyophilizer for 24 h, affording a product with mean yield of 86%, and the MW of 4900 by ^1^H NMR and of 5640 by *SEC*, named Fe_3_O_4_-*g*-PAA_68_ (the subscript figures of PAA represent the degree of polymerization by ^1^H NMR). Similarly, Fe_3_O_4_-*g*-PAA_130_ with mean yield of 85% was obtained when the molar ratio of Fe_3_O_4_@CPADB to AA of 1:150 was adopted. The experimental MW was determined to be about 9360 by ^1^H NMR, and the apparent MW was approximately 11,620 by *SEC*.

To synthesize the hybrid copolymer, MAEFC was first prepared as shown in Additional file 1: Figure S1. For synthesis of Fe_3_O_4_-*g*-PAA-*b*-PMAEFC, Fe_3_O_4_-*g*-PAA_68_ of 0.0404 g (0.0202 mmol) was added to a 25 ml Schlenk flask and entirely dissolved in 2 ml deionic water. Then, 1.3790 g (2.01 mmol) MAEFC and 0.0008 g AIBN (4.87 × 10^−3^ mmol) were added into a spotless beaker and completely dissolved in 8 ml DIOX before the mixed solution was placed in the flask. The flask was degassed via a successive freeze–pump–thaw cycle three times and ultimately suffused with N_2_. The reaction system was refluxed at 80 °C for 24 h with violent magnetic stirring. The crude product was repeatedly precipitated in *n*-hexane three times to remove excess reagents and unreacted monomer, and then dried in a lyophilizer for 24 h, giving a final product (mean yield: 78%), denominated P_2_. Similarly, the hybrid copolymers with molar ratios of Fe_3_O_4_-*g*-PAA_68_ to MAEFC of 1:50, Fe_3_O_4_-*g*-PAA_130_ to MAEFC of 1:100 and 1:200 were synthesized and named P_1_, P_3_ and P_4_, respectively (Table 1).

### Preparation of hybrid magnetic copolymer micromicelles

The hybrid copolymer micromicelles were prepared by a dialysis technique. In detail, 25 mg sample was thoroughly dissolved in 8 ml 1,4-dioxane with vigorous stirring, and then transferred into a dialysis bag with a molecular weight cutoff (MWCO) of 2000. The bag was directly immersed into 2000 ml deionized water to dialyze for 48 h. The water was replaced hourly for the first 3 h, and then once every 7 h. After dialysis, the micelle solution obtained was put into a 25 ml volumetric flask, and the maximal micelle concentration was 1 mg ml^−1^.

### Measurements and characterization

#### Chemical structural characterization

FT-IR spectra were recorded on an EQUINX55 Fourier transform infrared spectrometer (FTIR, Bruker Corp., Germany) using KBr pellets. ^1^H NMR analysis was performed on a Bruker Avance III 400 MHz NMR spectrometer (^1^H NMR, Bruker Corp., Germany, 400 MHz) using TMS as internal standard substance. X-ray diffraction (XRD) studies were performed on a D/Max-2550 VB+/PC X-ray diffractometer (Rigaku, Japan) employing Cu radiation at a voltage of 40 kV, a current of 30 mA and a scanning rate 10° min^−1^. Size/steric exclusion chromatography (*SEC*) was used to measure relative MW and PDI at a column temperature of 35 °C. The *SEC* system (EcoSEC, Tosoh Corp., Japan) was calibrated with linear polystyrene standards and THF as the eluent at a flow rate of 1 ml min^−1^. The dried samples were dissolved in THF at a concentration of 2 mg ml^−1^ and filtered through a 0.45 μm Teflon filter.

#### Physicochemical characterization of self-assembly micelles

The formation of micromicelles was studied through fluorescent spectrometry on a fluorescence spectrophotometer (PE LS55, PE Corp, USA) using pyrene as a fluorescent probe. Details were described in Additional file 1. The morphologies and sizes of the micelles were observed on a JEM-2100 transmission electron microscope (TEM, Electronics Corp., Japan) at an accelerating voltage of 200 kV. The surface morphologies were observed on a SU-8020 cold field emission scanning electron microscope (FESEM, Hitachi High-Technologies Corp., the Netherlands). Hydrodynamic diameters (D_*h*_) and size distribution were measured at room temperature by dynamic light scattering (DLS, BI-90Plus, Brookhaven Instrument Corp., USA) equipped with a He–Ne laser of wavelength of 660 nm, deflection angle of 90° and output power of 15 mW. Before measurement, the micelle solutions were dialyzed, and then diluted into a solution with a concentration of 1.0 mg ml^−1^. After the solution was filtrated through a 0.45 μm Millipore filter, the right amount of micellar solution was poured into a cuvette for measurement. The experiments were performed three times and the data were averaged. UV–vis spectra were recorded using a UV-3900/3900H UV–vis spectrophotometer (Hitachi, Japan). *Zeta* potentials (ξ) were measured by the laser particle *zeta* potential detecting instrument (Delsa Nano C, Beckman Coulter, USA) at 25 °C. Cyclic voltammetry (CV, CH Instrument Company, Shanghai, China) measurement was conducted with a conventional three-electrode cell and 0.05 M tetra-*n*-butylammonium hexafluorophosphate (CH_3_CH_2_CH_2_CH_2_)_4_N(PF_6_) as supporting electrolyte at ca. 25 °C and a scan rate of 50 mV s^−1^. The hysteresis loops were recorded on a vibration sample magnetometer (VSM, JDM-13, Lake Shore Corp, USA).

### Loading and in vitro release of drug

The hybrid polymer sample of 20 mg and PTX of 5 mg were added in 5 ml DMF. The solution was sufficiently stirred overnight to ensure thorough dissolution. Then, deionized water was dropwise added into the above solution until the solution turned into turbid, with uniformly stirring. After further stirred for about 2 h, the solution was transferred into a dialysis bag with MWCO 2000 for dialysis against 1000 ml deionized water for 48 h at room temperature. The dialysate was centrifugally separated at a rate of 500 rpm for about 10 min to remove the unloaded PTX, and then filtered through a 0.8 μm filter head. A solid powder was obtained by lyophilization and stored in a low-temperature environment for use.

To determine the loading content (LC) and encapsulation efficiency (EE) of drugs, the PTX-loaded micromicelles were redissolved in DMF to obtain the concentration of the PTX loaded in micromicelles (C) by monitoring the absorbance (A) of the solution at 210 nm using a UV–vis spectrometer (U-3900/3900H, Hitachi Corp., Japan):

2 $$ {\text{LC }}\left( \% \right) \; = \; \left( {{{\text{Mass of drug in micelle}} \mathord{\left/ {\vphantom {{\text{Mass of drug in micelle}} {\text{Mass of drug{-}loaded micelle}}}} \right. \kern-0pt} {\text{Mass of drug{-}loaded micelle}}}} \right)\; \times \; 100\% $$

3 $$ {\text{EE }}\left( \% \right) \; = \; \left( {{{\text{Mass of drug in micelle}} \mathord{\left/ {\vphantom {{\text{Mass of drug in micelle}} {\text{Mass of the added drugs}}}} \right. \kern-0pt} {\text{Mass of the added drugs}}}} \right)\; \times \; 100\% $$

The calibration equation used was as follows:

4 $$ C\left( {{\text{mg}}\, {\text{l}}^{ - 1} } \right) = 6 2. 6 2A - 1. 1 3 5 3 $$

For dual-stimuli responsive release experiments, 3 mg of the representative lyophilized drug-loaded P_4_ micromicelle (PTX content: 0.33 mg) was dissolved in 3 ml PBS or PBS containing oxidants. Then the solution was added into a dialysis bag with MWCO of 2500 and dialyzed against 250 ml PBS solutions of pH 7.4, 6.3 and 5.3 with and without H_2_O_2_, with continuous shaking at 100 rpm at 37 °C. At a given time, aliquots of 3 ml solution outside the bag was fetched out and replaced by the same volume of the corresponding release medium. As a control, the delivery of free PTX was also studied at pH 7.4 and cancer environments (pH 5.3 and H_2_O_2_ 0.8%) under the same conditions, especially an identical PTX amount to the PTX-loaded micromicelles (0.33 mg). The PTX amount released was calculated by monitoring the absorbance at 210 nm of the release medium using the calibration Eq. (4). The accumulative PTX release was estimated as follows:

5 $$ {\text{Cumulative PTX release}} \%  \; = \; {{{\text{M}}_{t} } \mathord{\left/ {\vphantom {{{\text{M}}_{t} } {{\text{M}}_{0} }}} \right. \kern-0pt} {{\text{M}}_{0} }}\; \times \; 100\% $$

where M_*t*_ and M_0_ stand for the amount of PTX at time *t* and the amount of PTX loaded in the micromicelles, respectively.

### In vitro cytotoxicity assay

The in vitro cytotoxicities of blank and PTX-loaded copolymer micromicelles were evaluated by a MTT assay as per methods reported elsewhere [55, 56]. Simply, A549 lung cancer cells were seeded into a 96-well plate at a density of 5 × 10^4^ cells well^−1^ and cultured 24 h in 200 μl of a complete Dulbecco’s modified Eagle’s medium (DMEM) containing 10% hyclone fetal bovine serum at 37 °C in 5% CO_2_ atmosphere for 24 h. Then, the culture medium was removed and cells were washed with PBS solution of pH 7.4. In the meanwhile, free PTX, blank and PTX-loaded copolymer micelle solutions with a range of concentrations were prepared in PBS solution of pH = 7.4, and another PTX-loaded micromicelle solution was prepared in PBS solution of pH 5.3 containing 0.8% H_2_O_2_, and added to the medium-removed 96-well plates. Then, 20 μl of each solution was added to the corresponding wells, followed by 24 h of incubation. After that, the medium was replaced by 200 μl of fresh DMEM. 20 μl of 5 mg ml^−1^ MTT stock solution was then added to each well. After 4 h, the supernatant was discarded, and the formazan crystals were dissolved in 110 μl DMSO for each well. The well plates were shaken for another 10 min at room temperature before measuring the absorbance at 490 nm with a 96-well universal microplate reader [Model 680, Bio-Rad laboratories (UK) Ltd.]. Cell viability (%) was calculated as previously described [56]. The Student’s *t* test was used to determine the significance of any pairs of observed differences. Differences were considered statistically significant when *p* < 0.05. All quantitative results are reported as mean values ± standard deviation from data obtained from at least three separate experiments.

### Stability of copolymer micromicelles with and without PTX

The copolymer micromicelles with and without PTX were stored in a refrigerator at 4 °C for 21 days. The stability was monitored by changes in the PTX concentration or/and particle size D_*h*_ during the storage period.

## Additional file

**Additional file 1.** The related preparation including Fe_3_O_4_ NPs and monomer MAEFC, modification, characterization, performance measurements and physicochemical data for supporting the manuscript.

## Abbreviations

Fe_3_O_4_ NPs: ferroferric oxide or iron oxide nanoparticles
RAFT: reversible addition-fragmentation chain transfer
Fe_3_O_4_-*g*-PAA-*b*-PMAEFC: Fe_3_O_4_ graft poly(acrylic acid)-*block*-poly(2-methacryloyloxyethyl ferrocenecarboxylate) block copolymers
FTIR: Fourier transform infrared spectroscopy
NMR: nuclear magnetic resonance
SEC: size/steric exclusion chromatography
XRD: X-ray diffraction
TGA: thermal gravimetric analysis
TEM: transmission electron microscopy
SEM: scanning electron microscope
FESEM: field emission scanning electron microscope
DDS: drug delivery systems
ROS: reactive oxygen species
ATP: adenosine-5′-triphosphate
APTES: (3-aminopropyl)triethoxysilane
Fe_3_O_4_@CPABD: Fe_3_O_4_ graft 4-cyano-4(thiobenzoylthio)pentanoic acid
CTAs: chain transfer agent
Fe_3_O_4_-*g*-PAA: Fe_3_O_4_ graft poly(acrylic acid)
PAA: poly(acrylic acid)
Cp: cyclopentadienyl
MAEFC: 2-methacryloyloxyethyl ferrocenecarboxylate
MW: molecular weight
Mn: number-average molecular weight
PDI: polydispersity index
D: average crystal size
CMC: critical micelle concentration
DLS: dynamic light scattering
D_*h*_: hydrodynamic diameter
*M*_s_: saturation magnetization
*H*_c_: negligible coercivity
*M*_r_: remanence values
ξ: *zeta* potentials
CV: cyclic voltammetry
E_p,anodic_: anodic oxidation potentials
ΔE: peak separation
*I*_p_: peak currents
ν^1/2^: the square root of the scan rate
Fc: ferrocene moieties
Vc: ascorbic acid
H_2_O_2_: hydrogen peroxide
NaClO: sodium hypochlorite
PTX: paclitaxel
MTT: 3-[4,5-dimethylthiazol-2-yl]-2,5-diphenyltetrazolium bromide
A549: human lung cancer cell lines
UV–vis: ultraviolet visible spectroscopy
FCA: ferrocene carboxylic acid
TCI: Tokyo Chemical Industry
HEMA: hydroxyethyl methacrylate
DCC: *N*,*N*′-dicyclohexylcarbodiimide
DMAP: dimethylaminopyridine
AA: acrylic acid
AIBN: 2,2′-azobis(isobutyronitrile)
CPADB: 4-cyano-4(thiobenzoylthio)pentanioc acid
HOBT: 1-hydroxybenzotriazole
EDC·HCl: 1-ethyl-3-(3-dimethyllaminopropyl)carbodiimide hydrochloride
DCM: dichlormethane
DIOX: 1,4-dioxane
C_16_H_36_F_6_NP: tetra-*n*-butylammonium hexafluorophosphate
C_2_H_3_N: acetonitrile
LC: loading capacity
EE: encapsulation efficiency
MWCO: molecular weight cut-off
DMEM: Dulbecco’s modified Eagle’s medium
C: the concentration of the PTX loaded in micromicelles
A: absorbance
M_*t*_ and M_0_: the amount of PTX at time *t* and the amount of PTX loaded in the micromicelles, respectively
