# Supplementary material for: Dual stimuli-responsive Fe3O4 graft poly(acrylic acid)-block-poly(2-methacryloyloxyethyl ferrocenecarboxylate) copolymer micromicelles: surface RAFT synthesis, self-assembly and drug release applications
Source: J Nanobiotechnology. 2017 Oct 27;15:76. doi: 10.1186/s12951-017-0309-y (PMC5658962; doi:10.1186/s12951-017-0309-y)
Supplement: Supplementary file 1 — Additional file 1. The related preparation including Fe3O4 NPs and monomer MAEFC, modification, characterization, performance measurements and physicochemical data for supporting the manuscript. [file 12951_2017_309_MOESM1_ESM.doc]

**Supporting information**

**Multiple stimuli-responsive Fe_3_O_4_ graft poly(acrylic acid)-*block*-poly(2 -methacryloyloxyethyl ferrocenecarboxylate) copolymer micromicelles: Surface RAFT synthesis, self-assembly and drug release applications**

Yuan Wang, Xue-Yin Zhang, Yan-Ling Luo*, Feng Xu*, Ya-Shao Chen, Yu-Yu Su

(Key Laboratory of Macromolecular Science of Shaanxi Province, School of Chemistry and Chemical Engineering, Shaanxi Normal University, Xi’an 710062, P. R. China)

Corresponding authors’ details:

**Yan-Ling Luo and Feng Xu**

Fax: +86 29 81530727.

E-mail addresses: luoyanl@snnu.edu.cn (Y. L. Luo) and fengxu@snnu.edu.cn (F. Xu).

Affiliation: Key Laboratory of Macromolecular Science of Shaanxi Province, School of Chemistry & Chemical Engineering, Shaanxi Normal University, Xi'an 710062, People's Republic of China.

**1.** **Preparation of** **magnetic Fe_3_O_4_ nanoparticles**

Ferric chloride hexahydrate (FeCl_3_·6H_2_O, analytically pure) and ferrous chloride tetrahydrate (FeCl_2_·4H_2_O, analytically pure) were provided by the Shanghai Macklin Biochemical Co., Ltd, China. To prepare magnetic Fe_3_O_4_ nanoparticles (NPs), a mixture of ferric (1.25 g, 6.29 mmol) and ferrous chlorides (2.50 g, 9.24 mmol) were dissolved in 20 ml deionized water in a 250 ml three-necked flask attached with a reflux condensation tube, and was mechanically stirred for 30 min under N_2_ to exclude O_2_. Then, ammonium hydroxide was dropwise added into the mixture solution with a constant pressure funnel within about 30 min to adjust the solution pH to 11. The reaction solution was heated to 50 °C for 6 h under N_2_ atmosphere, accompanying with vigorous mechanical stirring at a rate of 1000 rpm. The crude product was cooled in an ice bath by centrifuging at 4000 rpm, and washed several times with water and ethanol to remove excess chloride anions and iron cations. The precipitates were dried in a vacuum oven at 40 °C to afford black solid powder, magnetic Fe_3_O_4_ NPs (Mean yield: 75%).

**2. Synthesis of** **APTES-modified magnetic Fe_3_O_4_ NPs**

Typically, magnetite Fe_3_O_4_ NPs (1.0 g) was dispersed into 100 ml absolute ethyl ethanol (CH_3_CH_2_OH, 99.5%, Sinopharm Chemical Reagent Co. Ltd.) in a 250 ml four-necked flask, and ultrasonicated for about 1 h. Then, 20 ml ammonium hydroxide (NH_3_·H_2_O, 25*wt*%, Sinopharm Chemical Reagent Co. Ltd.) was added into the the mixture and ultrasonicated for another 20 min. Under mechanically stirring, APTES (99%, Aladdin Industrial Corp., Shanghai, China) of 8 ml was added into the above mixture. The reaction mixture was heated to 60 °C, and the reaction was allowed to proceed at a stirring rate of 1000 rpm for 48 h under N_2_ atmosphere. Impured product were separated from the reaction solution by centrifuging at 4000 rpm, and alternately washed with ethanol and deionized water svseral times until neutrality to excess ammonium ions. The pure nut-brown precipitates were dried in a vacuum oven at 40 °C for 24 h to give APTES-modified magnetite nanoparticles. The **amino** content on the surface of the modified Fe_3_O_4_ NPs was determined by potentiometric titration to be 18.25 **m**mol·g^-1^.

**3. Determination of amino contents and calculation of the surface coverage**

The **amimo** content on the surface of the APTES-modified magnetic Fe_3_O_4_ nanoparticles was determined by potentiometric titration as per references [1,2]. Before measurements, hydrochloric acid (HCl) and sodium hydroxide (NaOH) standard solutions were prepared and titrated through acid-base titration methods, giving a HCl and NaOH concentration of 0.0943 and 0.1073 mol L^-1^, respectively. To determine the **amimo** content, the APTES-modified magnetic Fe_3_O_4_ nanoparticles with mass m_0_ grams were fetched and placed in a 250 ml conical flask. Then, a definite amount of HCl standard solution (n_1_, mol) was added and ultrasonically oscillated until the mixture retained uniform. The APTES-modified Fe_3_O_4_ HCl solution was rapidly titrated with the above NaOH standard solution having known concentrations (c_2_, mol L^-1^). The change of pH values with alkali volume (V) was recorded and the pH-V curve was made. The corresponding V value on the curve at the time of the titration end point is the actual consumption of the alkali volume (V_e_, ml). The amino content (ω, **m**mol·g^-1^) was calculated in line with the formula (1) as follows:

ω=(n_1_-c_2_V_e_)/m_0_ (1)

In this work, the **amino** content on the surface of the modified Fe_3_O_4_ nanoparticles was determined to be about 18.25 **m**mol·g^-1^. **This corresponds to about 4.23 APTES molecules per Fe_3_O_4_ NP covering on the surface of the Fe_3_O_4_ NP, or the extent of the APTES particle coverage is about 2.03×10^-12^ mol cm^-2^.**

**To compare the actual coverage with the theoretical value, the molar volume of APTES molecules is obtained through multiple routes including the calculation from the data of its molar mass and density, and via the computational approach of quantum chemistry at a B3lyp/6-31G(d,p) level** [3]**; this value is about 235.5 cm^3^ mol^-1^. Suppose that the APTES molecules are considered as sphericity and arrange on the surface of Fe_3_O_4_ NPs in a monomolecular layer, the radius of a APTES molecule can be calculated to be ca 4.54×10^-8^ cm (0.454 nm), and its projected area is about 6.48×10^-15^ cm^2^ (0.648 nm^2^). Considering the principle of close packing of spheres, the space occupancy of the APTES molecules is about 0.9069 or the area occupied by a APTES molecule should be 1.1026πr^2^. The actual projected area is about 7.15×10^-15^ cm^2^.**

**It has been determined by XRD that the mean size of Fe_3_O_4_ NPs is about 10.5 nm, and thus the surface area of a Fe_3_O_4_ NP can be calculated to be about 346.2 nm^2^, viz., 3.46×10^-12^ cm^2^. Theoretically, there should be 484 APTES molecules covering on the surface of a Fe_3_O_4_ NP, viz., a surface coverage of 2.32×10^-10^ mol cm^-2^.**

**4. Preparation and characterization of monomer MAEFC containing ferrocenes**

2-methacryloyloxyethyl ferrocenecarboxylate (MAEFC) was synthesized through the esterification reaction between FCA and HEMA. Typically, in a hermetic 500 ml three-neck flask, FCA (9.20 g, 39.94 mmol), DMAP (4.8 g, 39.94 mmol) and HEMA (6.8 ml, 42.58 mmol) were dissolved in dried DCM (300 ml) under stirring for 60 min. The mixture was cooled to 0 °C for N_2_ atmosphere and stirred for about 60 min. Then DCC (10 g) was dissolved in desiccative DCM (60 ml) and dropwise added to the above solution within 30 min. The reaction was terminated after 24 h at room temperture, and at the same time the reaction mixed solution was filtered to remove 1,3-dicyclohexylurea (DCU). The filtrates were extracted several times with saturated NaHCO_3_ and deionized water in order to remove redundant DMAP, HEMA and FCA. The purified product was obtained through silica gel column chromatography using ethyl acetate and petroleum ether (*v/v*=1:9) as eluent. The orange MAEFC was obtained by rotary evaporation and drying in a vacuum oven at 40 °C (*Mean yield*: 75%).

Synthesis scheme of monomer MAEFC containing ferrocenes is shown in Figure S1(a), and FTIR spectrum is presented in Figure S1(b). It is seen that MAEFC produces characteristic vibration bands at 3038-3100, 2851-2947, 1706, 1620-1640, 1149-1283, 1014, 770-827 and 500-550 cm^-1^, which are assigned to the =C-H stretch vibration bands (terminal double bonds and Cp rings), C-H, C=O, C=C, C-O, the skeleton characteristic peak of Cp rings, =C-H bending vibration modes in ferrocene rings and Fe-C or Cp-Fe stretch modes [4], respectively, preliminarily indicating successful synthesis of monomer MAEFC. Figure S1(c) shows ^1^H NMR spectra of monomer MAEFC containing ferrocenes. The chemical shift signals reflecting ferrocene structural features are located at δ=4.83, 4.42 and 4.21 ppm; the proton shift signals of two methylene groups appear at δ=4.42 and 4.48 ppm (*t*, -O-*CH_2_-CH_2_*-O-), where the two signals at δ=4.42 ppm are overlapped ascribed to the ferrocene and methylene. The hydrogen proton resonance signals assigned to the terminal hydrogen of double bonds (-OOC(CH_3_)=*CH_2_*) emerge at δ=5.64 and 6.21 ppm, and the shift signal at δ=2.00 ppm is attributed to the methyl proton characteristic (-OOC(*CH_3_*)=CH_2_). These findings suggest that the monomer containing ferrocenes (MAEFC) is successfully synthesized in this work [5,6].

Figure S1 Synthesis scheme (a), FTIR (b) and ^1^H NMR spectra (c) of monomer MAEFC containing ferrocenes.

(a)


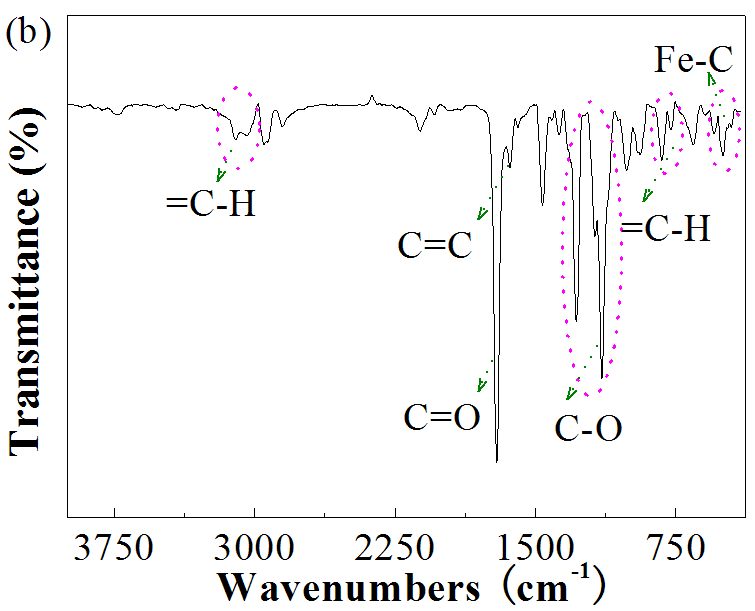


**5. TGA analyses**

**To investigate the relative amount of Fe_3_O_4_ in hybrid copolymers and the thermostability of hybrid copolymers, thermal gravimetric analysis (TGA) was carried out on a Q1000DSC+LNCS+FACS Q600SDT thermoanalyzer (TA Corp, USA) under N_2_ at a heating rate of 10 °C min^-1^. Representative TGA traces are shown in Figure** S2**. Pure Fe_3_O_4_ and APTES-modified Fe_3_O_4_ NPs have high thermostability, and only about 6.9% mass loss until 780 ºC.** **Fe_3_O_4_-g-PAA shows three obvious mass loss stages: about 16.2% loss below 210** **ºC is mainly assigned to the absorbed water, volatile solvents or impurity molecules during preparation and storage of the sample. In the second stage from 210 to 320** **ºC, PAA fragments start to be** **decarboxylated, and the loss reaches ca 23.0%. The mass loss from 320 to 780 ºC is ascribed to the degradation of PAA backbones. The residual weight of Fe_3_O_4_-g-PAA is approximately 7.0%, which should be assigned to Fe_3_O_4_ residues. Therefore, the grafting percentage of PAA on the surface of Fe_3_O_4_ is estimated to be about 86%, and the Fe_3_O_4_ content is approximately 7%. In comparison with Fe_3_O_4_-*g*-PAA, Fe_3_O_4_-*g*-PAA-*b*-****PMAEFC exhibits increased thermostability, and starts to decompose until 250 ºC probably due to the** **π-π stacking of the ferrocenyl groups in the copolymers. The decarboxylation and the decomposition of the copolymer skeleton happen almost simultaneously. From 250 to 450 ºC, the mass loss attains about 79.5%, and 84.6% till 740 ºC. The residual weight (15.4%) is higher than that in Fe_3_O_4_-*g*-PAA because of Fe atoms in ferrocene groups (The theoretical content of Fe is about 17% in PMAEFC).**

Figure S2 TGA curves of (a) Fe_3_O_4_, (b) APTES-modified Fe_3_O_4_, (c) Fe_3_O_4_-*g*-PAA_68_ and (d) Fe_3_O_4_-*g*-PAA_68_-*b*-PMAEFC_39_ (P_2_).

**6. CMC determination and the related physicochemical characterizations**

The formation of micromicelles was studied through fluorescent spectrometry on a fluorescence spectrophotometer (PE LS55, PE Corp, USA) using pyrene as a fluorescent probe. Details were described in ‘Supporting information’. In detail, 10 μl of 1×10^-3^ M pyrene acetone solution was added to a 10 ml sample tube. After acetone was completely volatilized, 5 ml of the hybrid copolymer solution ranging from 1×10^-4^ to 2.0 mg ml^-1^ was separately added to the above tubes, with magnetic stirring overnight. The curves of the micelle concentration *vs.* peak intensity ratios at 384/373 nm (I_3_/I_1_) were determined at an excitation wavelength of 332 nm and a slit width of 10 nm. The critical micelle concentration (CMC) was got by the point of intersection of tangents of curve transition points.

Figure S3 Plots of *I*_3_/*I*_1_ intensity ratios *vs* logarithm of the concentrations of the hybrid copolymers in aqueous solutions (log C) from 1×10^-4^ to 2 mg ml^-1^.

The morphologies and sizes of hybrid copolymer micelles were observed on a JEM-2100 transmission electron microscope (TEM, Electronics Corp., Japan) at an accelerating voltage of 200 kV. The TEM samples were prepared as follows: the freshly-prepared micelle solutions with a concentration of 1 mg ml^-1^ were filtrated through 0.45 μm aqueous-phase filter membrane before they were evenly dripped on a copper grid with carbon support films and air dried at room temperature.

*Zeta* potentials (ξ) were measured by the laser particle *zeta* potential detecting instrument (Delsa Nano C, Beckman Coulter, USA) at 25 ^o^C. All the measurements were set at a fixed scattering angle of 90^o^, and simulated physiological temperature for duration of 10 min, and each measurement was repeated three times. An average value was obtained from the three measurements.

**7. Magnetic parameters**

| Types of samples | *M*_s_ (emu g^-1^) | *M*_r_ (emu g^-1^) | *H*_C_ (Oe) |
| --- | --- | --- | --- |
| Native Fe_3_O_4_ | 58.14 | 3.62 | 51.14 |
| P_1_ | 1.95 | 0.18 | 6.32 |
| P_2_ | 4.71 | 0.25 | 3.31 |
| P_3_ | 0.42 | 0.018 | 4.87 |
| P_4_ | 3.08 | 0.10 | 8.02 |

Table S1 Magnetic parameters of the native Fe_3_O_4_ nanoparticles and the related hybrid copolymers.

**8. Electrochemical behaviors**

Figure S4 Plots of the peak separation ∆*E* of typical P_3_ hybrid copolymer modified electrode against the logarithm of the scan rate (log *v*) in THF solution of 0.1M (CH_3_CH_2_CH_2_CH_2_)_4_N(PF_6_) at 25 ^o^C.

**9. UV-vis studies of P_3_ sample oxidized by NaClO**

Figure S5 UV-vis spectra of typical P_3_ sample showing reversible redox transition between ferrocene and ferrocenium (Concentrations: 0.25 mg ml^-1^) in DMF before and after NaClO oxidation with concentrations (%) of 0.0, 1.8, 3.6 and 5.9, and then reduction by ascorbic acid (Vc).

**10. Change in D*_h_* induced by oxidization and reduction**

| Oxidants | D*_h_* after oxidation, nm | PDI | D*_h_* after reduction, nm | PDI |
| --- | --- | --- | --- | --- |
| H_2_O_2_ | 545±52 | 0.251±0.035 | 370±45 | 0.261±0.045 |
| NaClO | 390±60 | 0.286±0.045 | 356±50 | 0.290±0.072 |

Table S2* Change in D*_h_* for the micromicelles induced by oxidization and reduction.

*The sample is P_2_, and its concentration is 1.0 mg ml^-1^ in deionized water, and the original size is 260 nm.

**11. XPS analysis**

**X-ray photoelectron spectroscopy (XPS) profiles were determined at room temperature on an AXIS ULTRA spectrometer (Kratos Analytical Ltd., Shimadzu Corp., Japan) by using a monochromatic Al-Kα X-ray source (1486.6 eV) at a voltage of 15 *k*V. A high-resolution spectrum was obtained in the peak region of photoemission for Fe 2p electrons, as shown in Figure** S6**. As the amount of iron in micromicelles is rather low, the XPS measurements of the Fe2p photoelectron emissions need to perform for a longer period of time. From the shape of the Fe2p area, we can verify the presence of ferrocene and ferricenium.**

**Figure** S6 **High-resolution XPS spectra of element Fe before and after oxidization.**

**12. Drug loading**

**Table S3 LC and EE values of the copolymer micromicelles**.

| **Samples** | **P_1_** | **P_2_** | **P_3_** | **P_4_** |
| --- | --- | --- | --- | --- |
| **LC (%)** | **10.3** | **9.4** | **12.1** | **11.0** |
| **EE (%)** | **43.7** | **38.6** | **51.5** | **45.8** |

**References**

[1] Wuhan University. Analtical chemistry experiment. 4th ed. Beijing: Higher Education Press; 2001.

[2] Hu, W. Synthesis of carboxy**li**c acid chelating agent and modification on the surface of nanometer Fe_3_O_4_. Thesis for Degree of Master, Wuhan: Hubei University; 2013.

**[3] Xu T, Wang WL, Yin SW, Wang. Y Evaluation of electronic polarization energy in oligoacene molecular crystals using the solvated supermolecular approach. *Phys. Chem. Chem. Phys.* 2017; 19: 14453-61.**

[4] Liu Y, Yang A, Liu B, Yin D. Synthesis and optimization of bromoacetylferrocene. *Speciality Petrochemicals (In Chin.)* 2015; 32: 32–5.

**[5] Xu F, Li H, Luo YL.** **Redox-responsive self-assembly micelles from poly(N-acryloylmorpholine-*block*-2-acryloyloxyethyl ferrocene-carboxylate) amphiphilic block copolymers as drug release carriers. ACS Appl Mater Interfaces** 2017**; 9: 5181–5192.**

**[6] Wang Y, Luo YL, Xu F, Chen YS, Tang W. Redox-responsive PAEFc-*b*-PDMAEMA amphiphilic block copolymer self-assembly micelles: Physicochemical properties and anticancer drug controlled release. *J. Ind. Eng. Chem.* 2017; 48: 66–78.**
